# Genome-wide analysis links emerin to neuromuscular junction activity in *Caenorhabditis elegans*

**DOI:** 10.1186/gb-2014-15-2-r21

**Published:** 2014-02-03

**Authors:** Cristina González-Aguilera, Kohta Ikegami, Cristina Ayuso, Alberto de Luis, María Íñiguez, Juan Cabello, Jason D Lieb, Peter Askjaer

**Affiliations:** 1 Andalusian Center for Developmental Biology (CABD), CSIC/JA/Universidad Pablo de Olavide, 41013 Seville, Spain; 2 Department of Biology and Carolina Center for Genome Sciences, University of North Carolina at Chapel Hill, 27599 Chapel Hill, NC, USA; 3 Center for Biomedical Research of La Rioja (CIBIR), 26006 Logroño, Spain; 4 Current address: Biotech Research and Innovation Centre, University of Copenhagen, 2200 Copenhagen, Denmark

## Abstract

**Background:**

Laminopathies are diseases characterized by defects in nuclear envelope structure. A well-known example is Emery-Dreifuss muscular dystrophy, which is caused by mutations in the human lamin A/C and emerin genes. While most nuclear envelope proteins are ubiquitously expressed, laminopathies often affect only a subset of tissues. The molecular mechanisms underlying these tissue-specific manifestations remain elusive. We hypothesize that different functional subclasses of genes might be differentially affected by defects in specific nuclear envelope components.

**Results:**

Here we determine genome-wide DNA association profiles of two nuclear envelope components, lamin/LMN-1 and emerin/EMR-1 in adult *Caenorhabditis elegans*. Although both proteins bind to transcriptionally inactive regions of the genome, EMR-1 is enriched at genes involved in muscle and neuronal function. Deletion of either EMR-1 or LEM-2, another integral envelope protein, causes local changes in nuclear architecture as evidenced by altered association between DNA and LMN-1. Transcriptome analyses reveal that EMR-1 and LEM-2 are associated with gene repression, particularly of genes implicated in muscle and nervous system function. We demonstrate that *emr-1*, but not *lem-2*, mutants are sensitive to the cholinesterase inhibitor aldicarb, indicating altered activity at neuromuscular junctions.

**Conclusions:**

We identify a class of elements that bind EMR-1 but do not associate with LMN-1, and these are enriched for muscle and neuronal genes. Our data support a redundant function of EMR-1 and LEM-2 in chromatin anchoring to the nuclear envelope and gene repression. We demonstrate a specific role of EMR-1 in neuromuscular junction activity that may contribute to Emery-Dreifuss muscular dystrophy in humans.

## Background

The nuclear envelope (NE) is an essential structure conserved in all eukaryotes that separates the nucleus from the cytoplasm [1]. It is characterized by the presence of two concentric lipid membranes, the outer nuclear membrane (ONM), which is continuous with the endoplasmic reticulum, and the inner nuclear membrane (INM). The two membranes are fused where large nuclear pore complexes create transport channels to control trafficking of molecules between the nucleus and the cytoplasm. Numerous membrane proteins are integrated in the membrane bilayer. In the ONM, membrane proteins interact with microtubules, actin filaments and other cytoplasmic components. In the INM of higher eukaryotes, membrane proteins interact with the nuclear lamina, a protein meshwork of intermediate filaments that serves as a scaffold to maintain the morphology of the nucleus. INM and nuclear lamina proteins also interact with chromatin [2].

The NE is an important player in a number of cellular functions, including maintenance of structural integrity of the nucleus, chromatin organization, transcriptional regulation, DNA replication, cell-cycle control, differentiation, nuclear migration, and apoptosis [1,3-5]. Reflecting these many functions, mutations in NE components have been associated with numerous human diseases. In particular, a predominant group of diseases linked to alterations in the NE is caused by mutations in genes encoding the nuclear lamins (*LMNA*, *LMNB1* and *LMNB2* in humans) and is referred to as laminopathies. Laminopathies include diverse pathologies, such as muscular dystrophies, cardiomyopathies, bone disorders, neurological diseases, lipodystrophies and premature aging [5,6]. Laminopathy mutations have also been identified in several genes encoding ONM and INM proteins. Emery-Dreifuss muscular dystrophy (EDMD) was initially linked to mutations in the *EMD* gene, which encodes the INM protein emerin [7], but can also be caused by mutations in *LMNA* and NE genes *SYNE1*, *SYNE2* and *TMEM43*[6]. EDMD develops during childhood and manifests as degenerative muscle atrophy with cardiac defects [6]. Although clinical investigations focus on muscle abnormalities in EDMD patients, mice lacking emerin were found to display minimal muscle dysfunction but diminished motor coordination [8], suggesting that emerin could have additional functions apart from regulation of muscle formation and integrity. Interestingly, differences between humans and mice in relative expression levels of emerin and lamina-associated polypeptide 1 (LAP1) were recently proposed to explain the absence of muscle dystrophy in emerin mutant mice [9]. Since most NE genes are expressed ubiquitously, a main challenge is to understand how laminopathy mutations result in tissue-specific phenotypes [5,6]. One proposed hypothesis argues that an alteration of the integrity of the NE - for example, by affecting the nuclear lamina or the connection of the NE with the cytoskeleton - could lead to a structural weakness and a decrease in the ability of the nucleus to resist mechanical stress [10-12]. This could be of special relevance in muscle and skeletal disorders. However, symptoms affecting others tissues, such as the nervous system, cannot be explained simply by mechanical problems. Instead, several studies indicate that the NE contributes to chromatin organization by direct interaction with chromatin factors. For instance, the INM LEM-domain-containing proteins (*L*AP2, *e*merin and *M*AN1) interact with the chromatin binding protein BAF (also known as BANF1), histone deacetylases as well as with the nuclear lamins [2,13,14]. The nuclear lamins also interact with chromatin remodelers involved in heterochromatin formation, such as heterochromatin protein HP1 or the NURD complex [15].

Analyses of different organisms and cell types have shown that the NE is mainly associated with heterochromatin and silenced DNA [16-19]. Consistent with this, alterations in the distribution of heterochromatin marks, such as histone H3 lysine 9 methylation (H3K9me) and H3K27me, have been observed in murine and human myoblast cells harboring mutations in the *LMNA* gene [20,21]. Mutations in NE proteins can also lead to a reduction of transcription of muscle differentiation genes [22]. Although this reduction could at least partially be due to the role of NE components in heterochromatin formation, it has also been proposed that INM proteins can sequester transcription factors to the nuclear periphery and impede their binding to target genes. For example, in humans and mice, emerin physically interacts with lmo7 and β-catenin, two transcription factors involved in muscle differentiation [23,24], whereas in humans LEMD3/MAN1 tethers Smads to the NE, thereby affecting connective tissue differentiation [25-27].

While the importance of the NE as a regulator of nuclear architecture and gene expression is becoming increasingly evident, the molecular mechanisms underlying this remain elusive. In particular, the contributions of individual NE proteins to tissue-specific functions are not well understood. Characterization of the chromatin domains that interact with the NE is required to decipher how the NE contributes to nuclear organization. Until now, most experiments have focused on cultured cells, whereas few studies have been performed on cells within intact organisms. We therefore decided to analyze the DNA associated with two components of the NE, lamin/LMN-1 and emerin/EMR-1, in whole adult *Caenorhabditis elegans*. Due to its amenability to genetic manipulations, *C. elegans* is particularly suitable to genomic analyses across several genotypes and developmental stages [28]. Our results show that both LMN-1 and EMR-1 are associated with lowly expressed genes. As expected, similar DNA profiles were observed for the two proteins, but we also identified elements bound by only one of the two. ‘EMR-1 only’ elements were enriched for muscle and neuronal genes, which became accessible to LMN-1 association when *emr-1* was deleted. Furthermore, we observed that EMR-1 acts redundantly with another LEM domain containing protein, LEM-2, to repress transcription, consistent with their functional redundancy during mitosis [29], development and myogenesis [30], and signaling [31]. Finally, we demonstrate that EMR-1, but not LEM-2, is required for proper neuromuscular junction activity. Together, this study demonstrates the importance of EMR-1 in the control of chromatin organization and gene expression of muscle and neuronal genes, thereby providing clues as to how defects in INM protein function can have tissue-specific consequences.

## Results

### Identification of chromatin anchored to LMN-1 and EMR-1 by DamID

To investigate the specific role that different components of the NE play in the control of chromatin organization and gene expression, we generated genome-wide interaction maps of the sole *C. elegans* nuclear lamina protein, lamin/LMN-1, and the inner nuclear membrane protein emerin/EMR-1, in adult *C. elegans* nematodes using DamID [32]. This technique is based on the *in vivo* expression of chimeras of the *Escherichia coli* DNA adenine methyltransferase (Dam) and a chromatin-interacting protein. Upon interaction of the Dam-fused protein with chromatin, adenines in the vicinity are methylated. These DNA regions are subsequently identified by microarray analysis or high-throughput sequencing. DamID has been successfully used in many organisms and has been demonstrated to reliably identify NE-associated sequences [17,19]. To minimize experimental variation, we created *C. elegans* strains containing single copy insertions of the chimeric transgenes in chromosome II (Figure S1A in Additional file 1; Materials and methods). We used the promoter of the heat shock-inducible gene *hsp-16.41*, and due to the requirement for only trace expression levels of the chimeric protein, performed the DamID experiments without induction [19]. Without heat shock, transgene expression is undetectable by immunofluorescence or western blot analyses: however, after heat shock, the nuclear peripheral localization of chimeric proteins was readily observed (Figure S1B, C in Additional file 1). We also generated a control strain expressing GFP::Dam (which freely diffuses throughout the nucleus; Figure S1D in Additional file 1) to normalize for local chromatin accessibility [19]. Under normal growth conditions, nematodes containing chimeric proteins were fertile and indistinguishable from wild-type (Bristol N2) animals. We conclude that the Dam-fusion proteins mimic the localization of their endogenous counterparts and their presence did not produce any apparent deleterious effects.

To investigate nuclear organization in differentiated cells we performed DamID on young adults. Thus, the DamID signals we describe in the sections below represent the mixture of cell types of which the nematode is composed. We isolated adenine-methylated DNA from three biological replicates for each strain (Dam::LMN-1, Dam::EMR-1 and GFP::Dam) and analyzed it on *C. elegans* whole-genome high-density tiling microarrays. We normalized the DamID signals using MA2C [33] (Materials and methods) and then all MA2C data were quantile normalized to facilitate comparison between strains. The replicas were highly correlated (Pearson correlation between replicates ranging from 0.88 to 0.98; Figure S2A in Additional file 1) and genomic profiles of LMN-1 and EMR-1 association were constructed by averaging the three replicates.

### Maps of LMN-1 and EMR-1 reveal global similarities and association with silent DNA

**Figure 1:**
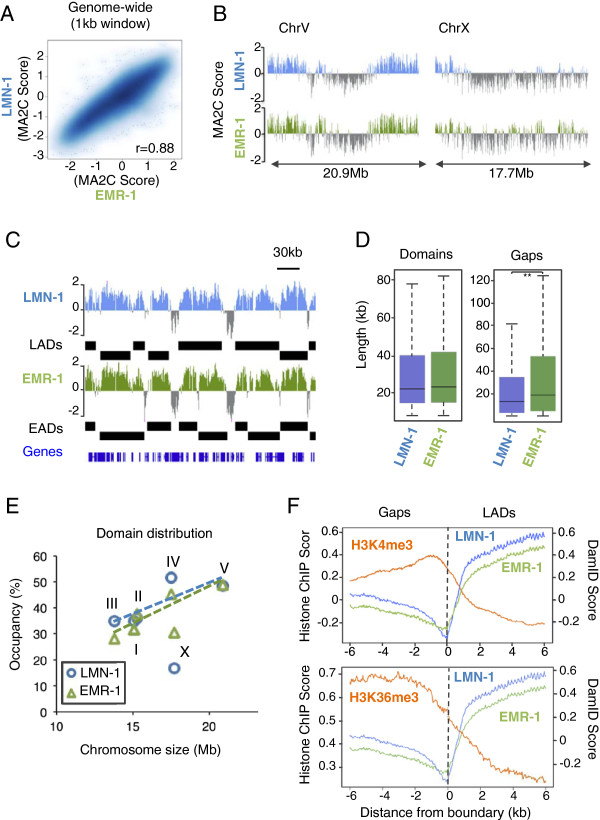
**LMN-1 and EMR-1 have overlapping DNA association profiles devoid of active chromatin marks. (A)** Genome-wide correlation plot of LMN-1 and EMR-1 occupancy. The correlation coefficient (r) is at the lower right corner. **(B)** Regions enriched for Dam::LMN-1 and Dam::EMR-1 in chromosomes V and X. For each track, the average MA2C score probe signal of three independent biological replicates is plotted. Other chromosomes are shown in Figure S3 in Additional file 1. **(C)** Representative region enriched for LMN-1 (MA2C score; blue) and EMR-1 (MA2C score; green) association. Positions of annotated genes are indicated below. LMN-1 and EMR-1-associated domains (LADs and EADs, respectively; black bars) were defined by a sliding window algorithm. **(D)** Sizes of LADs, EADs and regions between domains (Gaps). Bottom and top of boxes indicate the 25^th^ and 75^th^ percentiles, respectively, and lines in the boxes indicate medians. Whiskers indicate the lowest or the highest data point within 1.5× interquartile range from the box. Probability value from Wilcoxon rank sum test is indicated: **P < 10^-4^. **(E)** Relationship between chromosome size and LMN-1 or EMR-1 relative occupancy (sum of DamID domains divided by chromosome size). Linear regression lines for LMN-1 (blue; r = 0.82) and EMR-1 (green; r = 0.93) occupancy on autosomes are indicated. **(F)** Average H3K4me3 and H3K36me3 profiles at EAD boundaries. Chromatin immunoprecipitation (ChIP)-chip probe signal scores for indicated histone modifications are plotted. For comparison, averages of LMN-1 and EMR-1 DamID MA2C score are also shown.

The genome-wide profiles of Dam::LMN-1 and Dam::EMR-1 signals were very similar to each other (Pearson correlation of 0.88; Figure 1A). Both LMN-1 and EMR-1 associate preferentially with autosomal arms within 4 to 7 Mb of the telomeres. In contrast, the central part of the autosomes was largely devoid of LMN-1 and EMR-1 association (Figure 1B; Figure S3A in Additional file 1). Interestingly, the X chromosome showed a different pattern since interaction was mainly observed at the left end of the chromosome whereas only weak association was seen at the right arm. These patterns were similar to those of INM proteins LEM-2 [18] and LMN-1 [34] reported in embryos. Thus, the organization of autosomes within the nucleus is fundamentally different from that of the X chromosome in *C. elegans*.

To precisely define LMN-1-associated domains (LADs) and EMR-1-associated domains (EADs), we converted positive and negative DamID scores to +1 and -1, respectively, and used a window-based method to identify DNA regions continuously associated with LMN-1 or EMR-1 [18] (Materials and methods). Using a false discovery rate <5%, we identified 1,021 LADs and 1,106 EADs, each of which covers 37.5% and 37.8% of the genome, respectively (Figure 1C; Table S1 in Additional file 2). The size of LADs and EADs ranged from 8 to 300 kb with a median size of 22.7 kb and 23.9 kb for LMN-1 and EMR-1, respectively (Figure 1D). A large proportion of LADs and EADs was overlapped: 89% of DNA in LADs was also associated with EMR-1. LADs and EADs at the chromosome arms were separated by non-associated regions ('gaps'), which have a median size of 13.5 kb and 19.2 kb for LMN-1 and EMR-1, respectively (Figure 1D). It is important to note that genomic features, such as open reading frames, promoters and intergenic regions are found in both domains and gaps (Figure 1C).

Like LEM-2-associated regions [18], the fraction of DNA regions occupied by LMN-1 or EMR-1 in autosomes was positively correlated with the size of the autosomes (Figure 1E). In addition, we observed an equal or higher proportion of each autosome bound by LMN-1 than EMR-1. In contrast, the X chromosome showed a lower association with LMN-1 compared to EMR-1, suggesting that EMR-1 might play a distinct role in anchoring the X chromosome to the NE (Figure 1E; see below).

Previous studies in *C. elegans* embryos and other organisms have shown that the chromatin regions associated with the NE are marked predominantly by histone modifications characteristic of heterochromatin and are transcriptionally silent [17-19]. To investigate if this distribution is maintained in adult nematodes, we analyzed the levels of H3K4me3 and H3K36me3, both associated with transcriptional activity [35], in LADs and EADs. Sliding window analyses across EMR-1 domain boundaries showed that both LADs and EADs have lower H3K4me3 and H3K36me3 levels, and instead, gaps have higher levels of these two marks, suggesting that transcriptionally active chromatin is mainly localized in the nuclear interior in *C. elegans* adults (Figure 1F; Figure S3B in Additional file 1), as it is in embryos [18]. However, low H3K4me3 and H3K36me3 levels were not exclusively observed in LADs and EADs but also inside gaps, suggesting that not all chromatin away from the NE is transcriptionally active (Figure S3B in Additional file 1).

### Chromosome architecture is maintained during development

**Figure 2:**
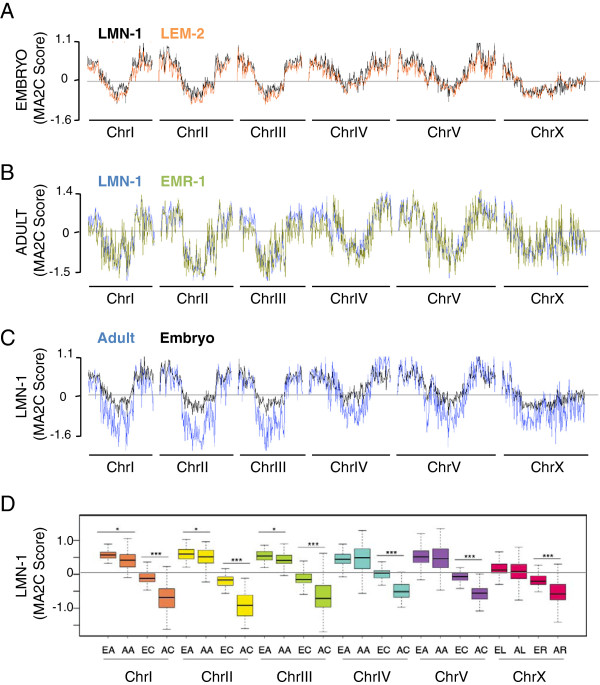
**Conservation of nuclear organization during development. (A, B)** Global association with chromatin is highly similar among NE proteins, both in embryos **(A)** for LMN-1 (black) and LEM-2 (orange) and during adulthood **(B)** for LMN-1 (blue) and EMR-1 (green). Embryonic LMN-1 DamID and LEM-2 ChIP MA2C scores were obtained from [34] and [18], respectively. **(C, D)** Representation of LMN-1 association as chromosomal profiles (**C**; embryos in black, adults in blue) and boxplot (**D**; as in Figure 1). **(D)** LMN-1 MA2C scores in embryos (E-) and adults (A-) averaged over chromosome arms (-A) and centers (-C), or, for the X chromosome, over the left (-L) and right (-R) portions. Probability values from Wilcoxon rank sum tests are indicated: *P < 0.05, ***P < 10^-11^. DamID signals at chromosome ends do not differ dramatically between the two developmental stages, whereas chromosome centers are devoid of LMN-1 interaction, particularly in adults. Probe scores were averaged in 100-kb windows for all panels.

Given the apparent similarity of chromosome organization in *C. elegans* embryos and adults, we analyzed in more detail the pattern of chromosomal associations with the NE in these two developmental stages. First, we compared the embryonic chromatin association profile of LMN-1 obtained by DamID [34] with the one of LEM-2 generated by chromatin immunoprecipitation (ChIP)-chip/seq [18]. The highly similar profiles cross-validate the two techniques (Figure 2A). Second, we also noted that the global profiles observed by LMN-1 and EMR-1 DamID during adulthood were very similar (Figure 2B), suggesting that the two DamID fusion proteins reliably detect chromatin at the NE. Finally, we compared LMN-1 DamID profiles in embryos and adults. In general, the boundaries between LMN-1-associated arms and non-associated centers coincided well in the two developmental stages. In addition, the dynamic range of LMN-1 association at chromosome arms was very similar between the two developmental stages (Figure 2C, D). However, in adulthood we observed slightly more NE association in the arms of chromosome IV, and during early embryogenesis higher association in the arms of chromosomes I, II and III (Figure 2C, D).

Interestingly, we found a clear difference in the behavior of the central part of the chromosomes. In early embryos, LMN-1 MA2C scores for the central part of the chromosomes were close to zero, while in adult nematodes LMN-1 association with chromosome centers was significantly lower (Figure 2C, D). Taken together, these data suggest that during adulthood segregation of chromatin into NE-associated and NE-excluded domains is more pronounced, especially at the central region of the chromosomes, which seems to be excluded from the nuclear periphery in most cells.

### Local differences between LMN-1 and EMR-1 DNA association reveal a specific role of EMR-1 in the regulation of muscle and neuronal genes

**Figure 3:**
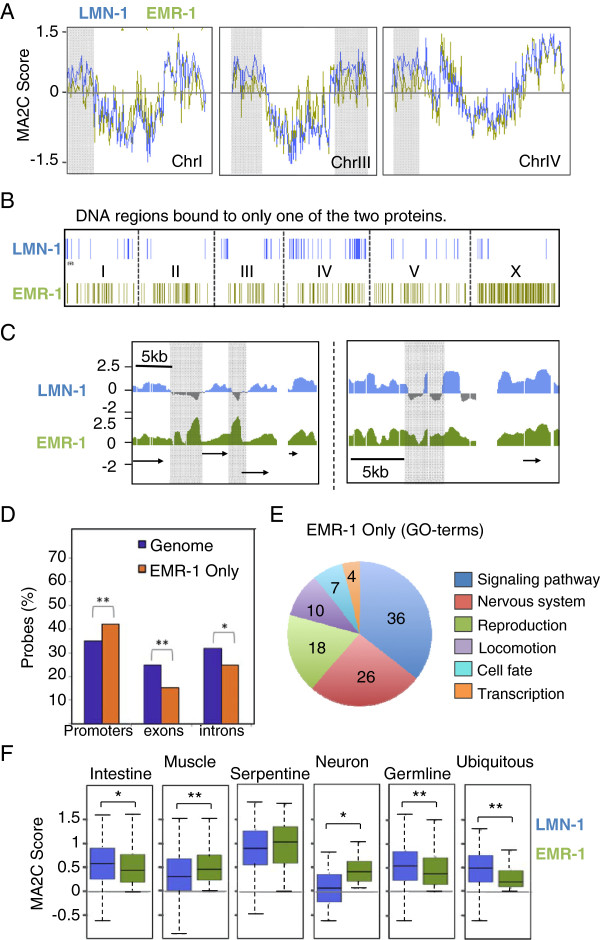
**EMR-1 associates with muscle and neuronal genes. (A)** Comparison of the regions enriched for LMN-1 and EMR-1 in chromosomes I, III and IV (100-kb window MA2C score). Grey boxes mark regions where LMN-1 associates more strongly than EMR-1. **(B)** Position of ‘LMN-1 only’ and ‘EMR-1 only’ elements as defined in Materials and methods. **(C)** Genome browser views of LMN-1 and EMR-1 occupancy in regions containing ‘EMR-1 only’ elements (grey boxes) at promoters (left panel) and in intergenic regions (right panel). **(D)** Distribution of ‘EMR-1 only’ probes in promoters, introns and exons relative to the genome. Probability values from one-sided binomial tests are indicated as in (B). **(E)** Gene Ontology (GO) analysis of genes included in ‘EMR only’ elements. GO terms with *P* < 0.05 were considered significant. Numbers in the pie chart indicate the number of genes in each category. **(F)** LMN-1 and EMR-1 occupancy for various gene sets defined by expression data. Number of genes: intestine (555), muscle (359), serpentine (325), neuron (14), germ line (618) and ubiquitous (174). Serpentine genes encode chemoreceptors expressed in a few neurons only. Boxplots (as in Figure 1) show the range of MA2C scores averaged over gene bodies. Probability values from Wilcoxon rank sum tests are indicated: *P < 0.05, **P < 10^-4^.

Despite the global similarities between LADs and EADs, there were also differences in the DamID profiles during adulthood. Specifically, the DamID signal of LMN-1 was higher than that of EMR-1 on the left arm of chromosomes I and IV and on both arms of chromosome III within regions covering 400 kb (Figure 3A). To perform a more detailed analysis of the differences in the chromatin association of LMN-1 and EMR-1, we subtracted the MA2C values obtained with the two Dam fusion proteins and plotted the resulting values (Figure S4A in Additional file 1). This revealed that areas of preferential association with LMN-1 were strongly enriched at autosome arms and at the left end of the X chromosome. Most likely, anchoring of these regions to the NE is mediated primarily by direct interaction with LMN-1 or by association with NE proteins other than EMR-1.

Although LMN-1 is ubiquitously expressed and physically interacts with EMR-1 [36], we also found elements associated exclusively with EMR-1 (Figure 3B, C). Contrary to LMN-1-enriched regions described above, these elements were more abundant close to the central part of the autosomes and on the X chromosome (Figure 3B; Figure S4 in Additional file 1). A systematic search identified 340 ‘EMR-1 only’ elements (Materials and methods) ranging from 339 bp to 3,328 bp and with a median size of 763 bp (Table S1 in Additional file 2). These elements were included in EADs and surrounded by LADs (Figure 3C; Figure S4B in Additional file 1). ‘EMR-1 only’ elements occurred significantly more often in promoters than expected, and occurred less often than expected in exons and introns (Figure 3C, D). Interestingly, Gene Ontology (GO) analyses of genes placed in or immediately downstream of EADs identified 29 GO categories, most of which were related to muscle and neuronal physiology, including signaling transduction at synapses (G-protein coupled receptor protein signaling pathway, *P* = 2.41e^-4^; synaptic transmission, *P* = 0.01; acetylcholine catabolic process, *P* = 0.04), behavior (behavior, *P* = 0.04), reproduction (reproductive process, *P* = 0.01) or locomotion (regulation of locomotion, *P* = 0.006) (Figure 3E; Table S5 in Additional file 3). Employing the same criteria used to defined ‘EMR-1 only’ elements, we identified 97 ‘LMN-1 only’ elements ranging from 406 bp to 2,097 bp and with a median size of 661 bp (Table S1 in Additional file 2). These ‘LMN-1 only’ elements were located almost exclusively in the large regions with high LMN-1/EMR-1 DamID ratios previously described (Figure 3B; Figure S4C in Additional file 1) and they were not enriched for genome features or GO term categories.

The identification of ‘EMR-1 only’ elements indicates that EMR-1 could have a predominant function in certain tissues. Indeed, we have observed that EMR-1 expression varies significantly between tissues (Figure S5 in Additional file 1). To further investigate whether LMN-1 and EMR-1 could play tissue-specific roles in chromatin organization, we analyzed LMN-1 and EMR-1 DamID scores of 6,954 genes that are expressed in a tissue-specific manner, as previously defined by others (Table S2 in Additional file 2; see Materials and methods for references). Of 6,954 genes, 4,909 (70.6%) were not anchored to the NE as reflected by the low MA2C values derived from DamID experiments performed in the whole animal (Figure S6A in Additional file 1). Importantly, in the 2,045 genes with a positive MA2C score (that is, anchored to the NE in most cells of the nematode), we observed significantly more association of intestinal, germline, and ubiquitous genes with LMN-1 than with EMR-1 (Figure 3F). In contrast, muscle and neuronal genes were associated with EMR-1 to a higher degree than they were with LMN-1 (Figure 3F; Figure S6B in Additional file 1), which is consistent with the GO analysis of ‘EMR-1 only’ elements. Our data thus suggest that EMR-1 is specifically involved in gene regulation in muscle and neurons.

### Lack of EMR-1 or LEM-2 produces local changes in nuclear organization

The unexpected finding of ‘EMR-1 only’ elements led us to speculate how they can be established. We imagine three scenarios for their formation: (1) EMR-1 is not only restricted to the NE but can diffuse into the nuclear interior; (2) EMR-1 localizes to patches of the NE devoid of LMN-1; or (3) EMR-1 is recruiting proteins to these DNA elements that inhibit access of LMN-1.

**Figure 4:**
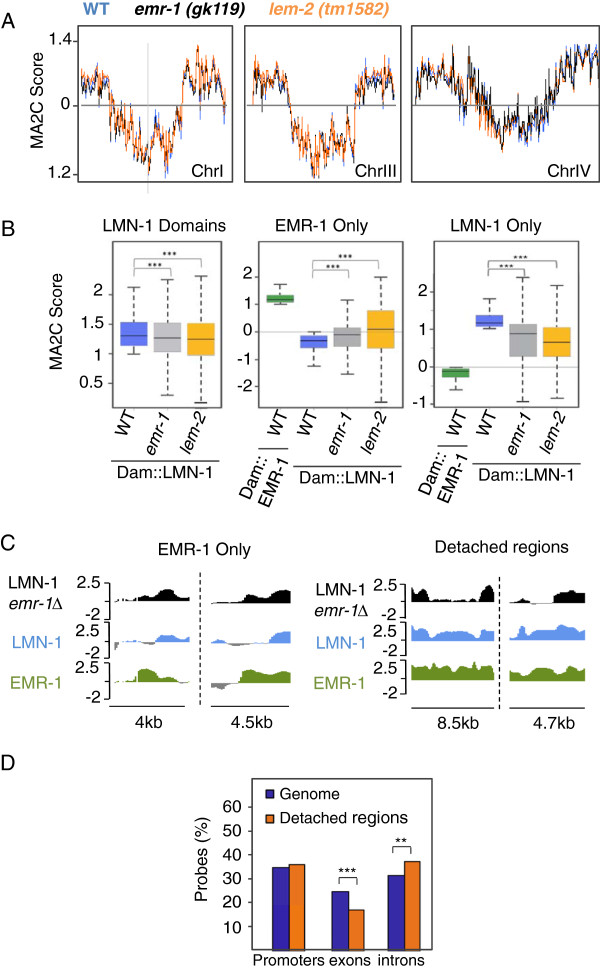
**Mutations in *emr-1 *or *lem-2 *produce local changes in nuclear organization. (A)** Comparison of the regions enriched for Dam::LMN-1 signals in chromosomes I, III and IV in wild type (WT, blue), *emr-1(gk119)* (black) or *lem-2(tm1582)* (orange) nematodes (100-kb window MA2C score). **(B)** Boxplots (as in Figure 1) of Dam::LMN-1 MA2C scores at probe level in LADs, ‘EMR-1 only’ or ‘LMN-1 only’ elements in wild type, *emr-1(gk119)* or *lem-2(tm1582)* nematodes. Dam::EMR-1 MA2C scores are included in the middle and right panels to illustrate the mutually exclusive association of ‘EMR-1 only’ and ‘LMN-1 only’ elements with EMR-1 and LMN-1 (***P < 10^-11^, Wilcoxon rank sum test). **(C)** Genome browser views of Dam::LMN-1 occupancy in wild type and *emr-1(gk119)* mutants. **(D)** Distribution of regions with reduced LMN-1 association in *emr-1(gk119)* mutants ('Detached regions') in promoters, introns and exons compared to the distribution of the genome. Probability values from one-sided binomial tests are indicated (**P < 10^-4^, ***P < 10^-11^).

We consider the first possibility unlikely because EMR-1, a transmembrane domain-containing protein, had never been visualized in the nuclear interior [37,38]. Although NE accumulation of EMR-1 is LMN-1-dependent [36], we cannot rule out the second possibility since biochemical purification of emerin from human HeLa cells identified protein complexes that lack lamins [39]. Moreover, small LMN-1-free patches of the NE involved in anchoring of ‘EMR-1 only’ elements would typically be undetectable at the resolution of light microscopy. To clarify whether EMR-1 could recruit additional factors to the ‘EMR-1 only’ elements, thereby sterically inhibiting Dam::LMN-1 access, we expressed Dam::LMN-1 in mutants homozygous for the *emr-1* null allele *gk119*[30]. If this hypothesis is true, in the absence of EMR-1, these elements should be able to interact with LMN-1. Based on the redundancy between EMR-1 and LEM-2 during embryogenesis [29] we also included the *lem-2*(*tm1582*) allele in our studies. Consistent with the absence of obvious growth phenotypes, mutations of *emr-1* or *lem-2* did not produce large-scale changes in LMN-1 distribution (Pearson correlation of 0.94 and 0.89 between wild type and *emr-1* and *lem-2* mutants, respectively; Figure 4A; Figure S7 in Additional file 1). However, in nematodes lacking EMR-1 or LEM-2, the ability of LMN-1 to interact with chromatin was mildly but significantly reduced, as can be seen by the lower MA2C score observed for LADs in these mutants (Figure 4B). In contrast, in both mutants, LMN-1 was localized more strongly to areas that were designated as ‘EMR-1 only’ in wild-type nematodes (Figure 4B, C). This effect was specific for ‘EMR-1 only’ elements since ‘LMN-1 only’ elements behaved like the rest of the LADs, with a mild and general decrease in association with the NE (Figure 4B). These data support the idea that EMR-1 (and LEM-2) could recruit transcription or chromatin remodeling factors that inhibit LMN-1 association with a subset of ‘EMR-1 only’ genomic locations.

Although lack of EMR-1 expression did not produce massive reorganization of the genome, we identified regions that were detached from the nuclear periphery upon EMR-1 deletion (Figure 4C; Table S1 in Additional file 2). These regions were specifically enriched in introns (Figure 4D) and GO analyses revealed that they were associated with genes involved in development of reproductive organs and muscles (Table S6 in Additional file 3).

### Depletion of EMR-1 and LEM-2 increases gene expression globally but reduces transcription of chromatin modifying enzymes

**Figure 5:**
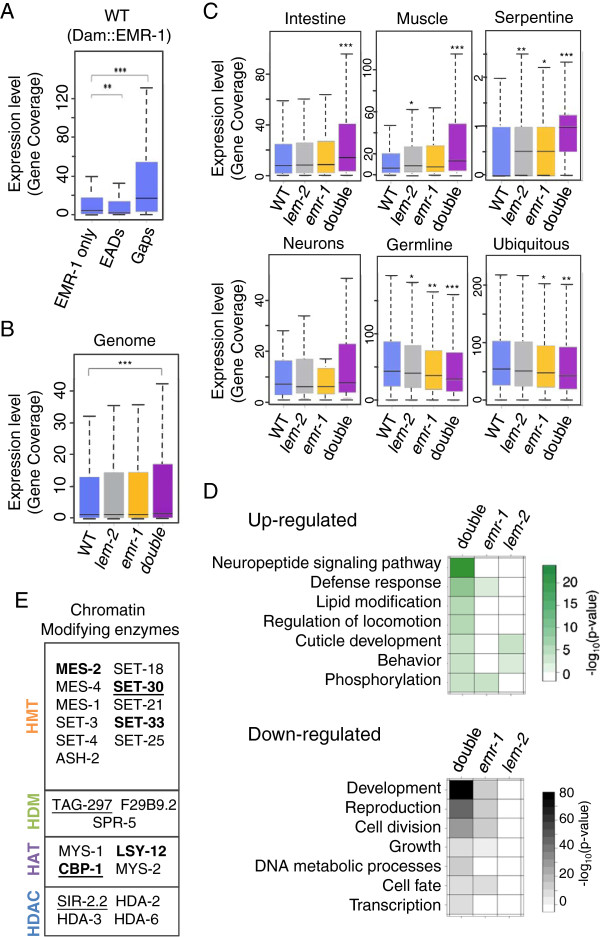
**Mutations in *emr-1 *and *lem-2 *de-repress gene expression. (A)** Boxplot (as in Figure 1) of expression levels of genes included in ‘EMR-1 only’ elements, EADs or gaps. Gene expression is measured as the median coverage of exons per gene. **(B)** Comparison of the expression of all genes in wild type (WT), *lem-2(tm1582)*, *emr-1(gk119)* and *emr-1(RNAi) lem-2(tm1582)* ('double') animals. **(C)** Expression of tissue-specific genes grouped as described in Figure 3. *P < 10^-2^, **P < 10^-4^ and ***P < 10^-11^. **(D)** Gene Ontology analysis of genes up- (green) or down-regulated (black) in *lem-2(tm1582)*, *emr-1(gk119)* and *emr-1(RNAi) lem-2(tm1582)* animals compared to wild type (*P* < 0.01). **(E)** Chromatin modifying enzymes with altered expression in *emr-1(RNAi) lem-2(tm1582)* nematodes. Most genes are down-regulated, except underlined genes, which are up-regulated. Genes also affected in the *emr-1*(*gk119*) single mutant are shown in bold.

The results above suggest that EMR-1 and LEM-2 may regulate the expression of certain gene classes. To test this directly, we performed RNA sequencing in wild-type animals and in animals depleted for EMR-1 and/or LEM-2. Data from wild-type nematodes confirmed that EMR-1 was associated with silent and poorly transcribed regions (Figure 5A). Similar results were obtained for LADs (Figure S8A in Additional file 1), supporting the notion of the nuclear periphery being a zone of generally repressed chromatin. Genes included in ‘EMR-1 only’ elements also showed reduced expression relative to genes in gaps. However, expression within ‘EMR-1 only’ regions was slightly but significantly higher than the expression across all EADs (Figure 5A).

Since LMN-1 DamID profiles of *emr-1* or *lem-2* single mutants did not reveal dramatic changes in LMN-1 domain organization, we analyzed gene expression in animals lacking expression of both EMR-1 and LEM-2. To bypass the embryonic lethality produced by the simultaneous deletion of EMR-1 and LEM-2, we knocked down *emr-1* expression by RNA interference (RNAi) in *lem-2* mutants. L1 larvae homozygous for the *lem-2(tm1582)* allele were fed with bacteria expressing *emr-1* double-stranded RNA. These nematodes developed to adulthood at the normal rate but produced a large number of dead embryos, which verified the effectiveness of the RNAi treatment [29]. As illustrated in Figure 5B, a general increase in RNA levels for all genes was observed in both singly and doubly depleted animals: in *lem-2(tm1582); emr-1(RNAi)* doubly depleted animals, the RNA levels of more genes were significantly altered relative to wild type (9.7% of protein-coding genes compared with 2.2% and 0.48% in *emr-1(gk119)* and *lem-2(tm1582)* single mutants, respectively; Figure 5B; Figure S8C in Additional file 1), most likely reflecting the redundant functions of EMR-1 and LEM-2.

Analysis of the genes that lose their NE interaction in *emr-1* mutants revealed a negative correlation between NE association and gene expression (Figure S8E in Additional file 1). Combined with the decreased association of chromatin to the nuclear periphery observed by LMN-1 DamID in the single mutants (Figure 4B), this result clearly indicates a role for EMR-1 and LEM-2 in gene repression. However, consistent with previous studies [34,40], we observed that not all the genes that were up-regulated in the *emr-1* mutant changed their position relative to the NE (Figure S8D in Additional file 1). This indicates that multiple processes influence the relationship between NE association and gene expression (see Discussion).

When we analyzed genes expressed in specific tissues, we also found that effects were maximal in the doubly depleted animals. Interestingly, an increase in expression of intestinal, muscle and serpentine genes was observed whereas germ line and ubiquitous genes were repressed (Figure 5C). Because the number of neuronal genes in this data set was very low (n = 14) we expanded our analysis to include 338 genes identified as pan-neuronal genes in larvae [41] (Table S2 in Additional file 2). Of these, 141 genes are associated with the nuclear periphery as judged from positive EMR-1 DamID MA2C scores. Interestingly, the pan-neuronal genes were significantly up-regulated in doubly depleted animals (Figure S6C in Additional file 1). These results were confirmed by GO analyses since GO categories related to the nervous system and locomotion were enriched among the significantly up-regulated genes (Figure 5D; Table S8 in Additional file 3). In contrast, down-regulated genes were associated with GO terms related to reproduction and basic cellular processes like cell cycle or transcription (Figure 5D). Intriguingly, genes related to chromatin organization were over-represented among the transcripts with reduced expression (*P* = 1.17e-6). In fact, the expression of 18 of the 38 chromatin modifying enzymes described in *C. elegans*[42] was significantly affected in the doubly depleted animals (47%, compared to 9.7% of all genes; Figure 5E; Table S8 in Additional file 3). We cannot rule out the possibility that some of the changes in expression levels of chromatin modifier enzymes could be secondary effects of chromatin reorganization upon EMR-1 and LEM-2 depletion. Nevertheless, these data support the idea that EMR-1 and LEM-2 could be especially important for regulating the activity of genes encoding chromatin-remodeling activities.

### EMR-1 is required for proper neuromuscular junction activity

**Figure 6:**
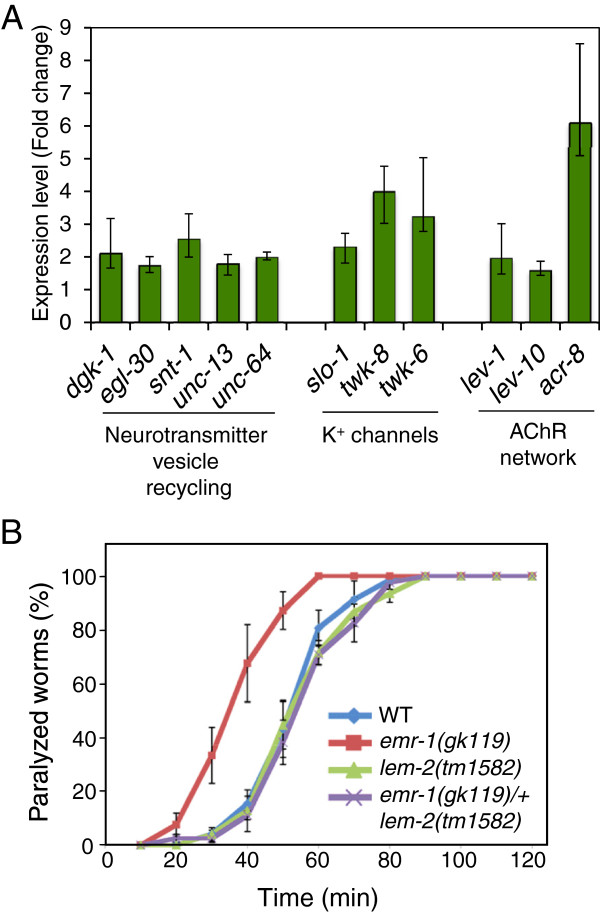
**Synaptic function is altered in *emr-1 *mutants. (A)** Fold increase in expression of selected genes involved in synaptic transmission in *emr-1(RNAi) lem-2(tm1582)* animals. Error bars indicate minimum and maximum values. **(B)** Aldicarb sensitivity experiments in wild type (WT), *emr-1(gk119)*, *lem-2(tm1582)* and *emr-1(gk119)/*+ *lem-2(tm1582)* mutants. Synaptic function was evaluated based on paralysis induced by the cholinesterase inhibitor aldicarb. Data points show the mean of four experiments and error bars indicate standard error of the mean.

Our results are in concordance with a role of emerin/EMR-1 and LEMD2/LEM-2 in muscle formation and activity as has been reported in various organisms [30,31,43]. However, to our knowledge, neither emerin nor LEMD2 has been implicated in neuronal processes. Since both NE tethering and expression of genes related to the nervous system and synaptic transmission were altered in *emr-1* and *lem-2* mutants (Figures 3, 4 and 5), we explored this possibility. Indeed, among the genes related to synaptic transmission, the expression of those encoding neurotransmitter vesicle factors, K^+^ channels, and acetylcholine receptor proteins was affected (Figure 6A). To investigate *in vivo* whether EMR-1 and LEM-2 play a role in neural physiology, we performed aldicarb sensitivity experiments to assess synaptic transmission activity. Aldicarb is a chemical analogue of the neurotransmitter acetylcholine and inhibits the activity of cholinesterase at neuromuscular synapses. Consequently, acetylcholine accumulates in the synaptic cleft, causing continuous activation of muscular acetylcholine receptors, muscle hyper-contraction and paralysis [44]. Alteration in aldicarb sensitivity can therefore be used to detect changes in acetylcholine release and perception.

We determined the time-course for aldicarb-induced paralysis in wild type, *emr-1* and *lem-2* single mutants and in *lem-2* nematodes heterozygous for the *emr-1* mutation (*emr-1*(*gk119*)/+; *lem-2(tm1582)*). We did not test for EMR-1/LEM-2 double depletion because RNAi is less effective in *C. elegans* neurons and the homozygous double mutants proceeding from heterozygous animals arrest during larval development [30]. Compared with wild-type animals, *emr-1* mutants were hypersensitive to aldicarb (50% of animals were paralyzed after approximately 35 minutes for *emr-1* mutants versus approximately 55 minutes for wild-type animals), whereas *lem-2* mutants retain normal aldicarb sensitivity (Figure 6B). Heterozygous *emr-1(gk119)/+* mutants were equally sensitive to aldicarb as wild-type animals, indicating that a single copy of *emr-1* is sufficient to maintain proper neuromuscular junction activity.

At this moment, we cannot distinguish whether the paralysis observed in *emr-1* mutants is due to changes in neurotransmitter release or muscle perception, or a combination of the two. Interestingly, *lem-2* mutants present alterations in muscle structure and function [30] and, unlike *emr-1* mutants, do not show altered aldicarb sensitivity. This indicates that EMR-1 and LEM-2 are not redundant in all their functions, making *C. elegans* a good model to study the tissue-specific roles of these two proteins.

## Discussion

A comparison of the genome-wide DNA association profiles of LMN-1, the only lamin protein in *C. elegans*, and of the INM protein emerin/EMR-1 indicates that EMR-1 defines two types of chromatin domains: a major type consisting of LADs and a minor type devoid of LMN-1 and enriched for genes expressed in specific tissues. LADs are conserved from embryos to adults and epigenetic marks suggest they are transcriptionally silent at both developmental stages (this study and [34]). In contrast, genes associated with EMR-1 only show higher expression and belong to functional categories that respond to stimuli. The difference between these two classes of chromatin domains might be mediated by posttranslational modifications of EMR-1 as well as other NE associated proteins. For instance, phosphorylation of human emerin has been shown to be important for the association with BAF [45,46].

The discovery of regions associated with EMR-1 but not with LMN-1 was surprising, since the two proteins physically interact at the NE and LMN-1 is required to localize EMR-1 properly [36]. Interestingly, although we could find ‘EMR-1 only’ elements in all chromosomes, they were especially abundant in the X chromosome. We do not know why the X chromosome has fewer interactions with LMN-1 compared to the autosomes, nor can we explain the particular enrichment of ‘EMR-1 only’ elements on the X chromosome. Our experiments were performed with *C. elegans* hermaphrodites, in which both X chromosomes undergoes approximately two-fold chromosome-wide transcriptional repression to achieve dosage compensation [47]. It is possible that the interaction with EMR-1 or other INM proteins could be important for this transcriptional repression. Our data suggest that the anchoring of the X chromosome to the NE could be more pronounced than previously inferred from LEM-2 ChIP data [18] or by LMN-1 DamID ([34] and this study).

We found that ‘EMR-1 only’ elements were surrounded by chromatin interacting with both LMN-1 and EMR-1. This indicates that ‘EMR-1 only’ elements are located at or very close to the NE, but raises the question as to why LMN-1 is not able to bind to these chromatin regions. Given that the absence of EMR-1 and/or LEM-2 allows the ‘EMR-1 only’ elements to interact more frequently with LMN-1, we hypothesize that EMR-1 and LEM-2 physically prevent LMN-1 from binding to these regions. It is known that human and mouse emerin interacts physically with transcription factors involved in muscle development, including β-catenin [23] and Lmo7 [24], and with chromatin associated factors, such as BAF and HDAC3 [13,48]. Similarly, the mammalian LEM-2-like protein MAN1 interacts with Smads at the NE to regulate transforming growth factor-β signaling [49]. *C. elegans* EMR-1 and LEM-2 may also associate with DNA-binding proteins and such protein complexes may hinder LMN-1 from binding to ‘EMR-1 only’ elements.

The NE is generally associated with repressed DNA and several of our observations suggest that EMR-1 and LEM-2 may also be involved in anchoring of silent heterochromatin to the nuclear periphery in specific tissues. First, genes within EADs are less expressed than genes in gaps. Second, we have demonstrated that, during adulthood, depletion of EMR-1 and/or LEM-2 caused a partial release of LADs from the NE and increased transcription of several hundred genes. These results are consistent with the delocalization and overexpression of repetitive heterochromatic transgene arrays observed upon simultaneous depletion of EMR-1 and LEM-2 in adult nematodes [50]. Interestingly, although chromatin associated with EMR-1 is less transcribed than chromatin found in gaps, genes in ‘EMR-1 only’ elements are significantly more expressed than genes in other EMR-1 domains. It is possible that the accumulation of transcription factors around ‘EMR-1 only’ elements might poise these regions for a rapid response to environmental stimuli. Supporting this idea, ‘EMR-1 only’ elements are enriched for GO categories related to signaling pathways and transcription. However, we have also noticed that gain or loss of NE anchoring do not always correlate with changes in gene expression. This has also been observed in other organisms. For instance, approximately one-third of genes that move towards or away from the NE during mouse embryonic stem cell (ESC) differentiation do not change transcriptional activity [40]. Similarly, changes in expression of genes that gain or lose lamin interaction after ecdysone treatment is marginal in *Drosophila* Kc cells [19]. Finally, mouse ESCs lacking B type lamins show altered transcription profiles but the alterations do not correlate with lamin B1 association in wild-type ESCs [51]. The reason for this lack of concordance between NE anchoring and silencing is not clear. Peric-Hupkes and colleagues [40] proposed that genes that move away from the NE during ESC differentiation are 'unlocked' to become activated at later steps of development. Likewise, results from a recent study in human adipose stem cells, indicate that dissociation of promoters from the nuclear lamins produces chromatin changes that enhance transcriptional permissiveness but do not necessarily elicit gene activation [52]. The molecular details of these intermediate states are unknown. The observation in *C. elegans* that the sequential action of two H3K9 methyltransferases promotes first anchoring of DNA to the NE and afterwards transcriptional repression has provided mechanistic insight into the uncoupling of the two processes [34].

Our data also indicate that genes specifically expressed in muscles and neurons are enriched in EADs. Furthermore, expression levels of genes related to locomotion and behavior were affected in *emr-1* and *lem-2* mutants. Several studies on Emery-Dreifuss muscular dystrophy patients and model organisms support the role of emerin and LEMD2 in muscle integrity, but their potential role in neurons had not been explored. Here we show that in *C. elegans*, localization and expression of genes related to neurotransmitter recycling, acetylcholine receptor network and ion-channels are affected in *emr-1* and *lem-2* mutants. Interestingly, neuromuscular junction activity is strongly affected in *emr-1* but not in *lem-2* mutants, indicating that the two genes are not redundant in all their functions. The role of emerin in neuronal activity may not be restricted to *C. elegans* since *emerin* knockout mice suffer relatively mild muscle defects and altered motor coordination [8]. In rodents, expression of emerin but not LEMD2 has been detected in neuroretinal cells, while both proteins are equally present in muscles [53]. Moreover, mutations in several other NE components, such as lamin A, BAF, lamin B1, and the LINC complex (which connects the NE to the cytoskeleton), are associated with neuropathies affecting both the peripheral and central nervous system [54-58]. Future analyses are needed to investigate whether emerin also contributes to proper neuronal physiology in humans.

Finally, we have demonstrated that, in adult nematodes, the NE-associated heterochromatin domains are highly enriched at the ends of autosome arms and at the left arm of chromosome X. This configuration is very similar to the one observed during embryogenesis [18,34]. However, we also found differences in NE-genome associations during development. These include few local changes in LADs placed in the chromosome arms and a general decrease in association of LMN-1 with the central part of the chromosomes in adults. Developmental changes in chromatin distribution have been observed in several organisms. The location of integrated small transgene arrays has been reported to be developmentally regulated, being randomly positioned in embryonic nuclei and accumulated in the nuclear interior in larvae [59]. The integration sites of these transgene arrays are unknown but their repositioning away from the NE coincides with transcriptional activation [59]. In murine embryonic stem cells, approximately 10% of genes change their interaction with the nuclear lamina during *in vitro* differentiation [40]. Together, these results suggest that, across evolution, global nuclear organization is established early in development and only a few specific regions change their localization in response to developmental requirements or changes.

## Conclusions

The comparison of chromatin associated with the conserved NE proteins LMN-1/lamin and EMR-1/emerin presented here reveals a large overlap between LMN-1- and EMR-1-associated domains but also the existence of numerous elements that interact with EMR-1 only. Muscle and neuronal genes are enriched in these ‘EMR-1 only’ elements and are deregulated in animals depleted for EMR-1 and LEM-2. Moreover, differential expression of EMR-1 and LEM-2 was observed across tissues.

The requirement for EMR-1 for the proper spatial and transcriptional control of muscle and neuronal genes demonstrated here prompts the idea that variation in NE composition among tissues may contribute to the correct adaptation of the organism to the environment. Combined with the defects in neuromuscular junction activity in *emr-1* mutants reported here, we propose that development of Emery-Dreifuss muscular dystrophy may involve altered nuclear architecture and gene expression, especially in muscles and neurons.

## Materials and methods

### Strains and plasmids

Strains carrying a single-copy insertion of Dam-fusion constructs were made using the MosSCI technique [60]. Strain and plasmid construction are described in Additional file 4.

### Immunofluorescence and western blot

Nematodes grown at 20°C were heat-shocked 1 h at 33°C and left to recover for 2 h at 20°C. Embryos were obtained and processed as described [61]. Anti-MYC primary antibody (Sigma-Aldrich, catalog number C3956, St. Louis, MO, USA) was diluted 1:100 for immunofluorescence and 1:400 for western blotting. Secondary goat anti-rabbit Alexa Fluor 633-conjugated antibody (Invitrogen, catalog number A21071, Carlsbad, CA, USA) diluted 1:1,000 and peroxidase-conjugated antibody (Sigma-Aldrich, catalog number A0545) diluted 1:5,000 were used for immunofluorescence and western blotting, respectively.

### DamID experiments

DamID experiments were performed using three biological replicates and all cultures were grown in parallel. DamID strains were synchronized from eggs prepared by hypochlorite treatment of gravid adults fed with OP50 *E. coli* and left to hatch overnight at 20°C in S-medium. Then, from each strain, approximately 35,000 L1s were grown in 50 ml S-medium containing GM119, a Dam^-^*E. coli* strain (F^-^*dam-3 dcm-6 metB1* galk2 *galT22 lacY1 tonA31 tsx-78 supE44 mtl-1*). This food source was used to avoid contamination by methylated *E. coli* DNA. Cultures were grown with continuous agitation (180 rpm) at 20°C for 53 h. At this time point the cultures were highly enriched for non-gravid young adults. Nematodes were frozen at -80°C until further processing.

Methylated genomic DNA (gDNA) was purified and amplified from 30 mg nematodes using a protocol based on [62] with some modifications. Briefly, 2.5 μg of gDNA isolated with DNAeasy kit (QIAGEN, Venlo, Limburg, Netherlands), was digested with 10 units *Dpn*I (New England Biolabs, Ipswich, MA, USA) overnight in 10 μl to cut at G^m^ATC sites. *Dpn*I was inactivated (80°C, 20 minutes) and then gDNA was ligated to double-stranded adaptors with 5 units T4 DNA ligase (5 U/μl; Roche, Basel, Switzerland) in a volume of 20 μl. After inactivation of the ligase (65°C, 10 minutes), DNA fragments were digested with 5 units *Dpn*II (New England Biolabs) in a final volume of 80 μl to cut non-methylated GATC sites, thereby preventing PCR amplification of non-methylated gDNA. Methylated DNA was amplified using adaptor-specific primers. PCR reactions were performed using 1 μl PCR Advantage enzyme mix (50×; Clontech, Otsu, Shiga, Japan), 10 μl of *Dpn*II digestion and the following program: (1) 68°C for 10 minutes, (2) 94°C for 1 minute, (3) 65°C for 5 minutes, (4) 68°C for 15 minutes, (5) 94°C for 1 minute, (6) 65°C for 1 minute, (7) 68°C for 10 minutes, (8) go to step 5 3×, (9) 94°C for 1 minute, (10) 65°C for 1 minute, (11) 68°C for 2 minutes, (12) go to step 9 20×. To achieve sufficient material, two PCR reactions from the same *Dpn*II digestion were pooled and purified with Qiaquick PCR purification kit (QIAGEN). Amplified Dam-methylated DNA was labeled and hybridized by the Roche Nimblegen Service Laboratory. Dye orientation of experiments is indicated in Table S4 in Additional file 2.

### DamID data processing

Nimblegen 2.1 M whole genome tiling arrays, with 50 bp probes, designed against WS180 (ce5) genome assembly, were used for all experiments. Data were analyzed with MA2C [33], which was used to normalize the log_2_ ratio (log_2_ [Dam::fusion/GFP::Dam control]) of each probe based on the probe behavior estimated by its GC content, and then to smooth the value by assigning the median across sliding windows of 300 bp. The resultant values are MA2C scores. Subsequently, data were subjected to quantile normalization over all available replicates and backgrounds to facilitate comparison between strains. Finally, the mean of normalized MA2C scores from the three independent biological replicates was calculated. We then converted the chromosome coordinates to WS190 (ce6) genome assembly using the Lift-Over tool from the UCSC genome browser [63] and used these values for the subsequent domain calling and sliding window analyses. In some cases, to facilitate chromosome-scale data visualization by reducing the number of data points, MA2C scores were averaged within non-overlapping 100 kb windows across the genome as indicated in the figure legends.

Sliding window analysis was performed using the left boundaries corresponding to the left edges of all EMR-1 domains (Table S1 in Additional file 2). Both sides of boundaries and sliding window analyses with LMN-1 boundaries provided the same results. Boxplots for gene classes were generated by averaging MA2C scores of probes included in regions between the transcription start site and end site. Coordinates for these sites were obtained from the UCSC genome browser (WS190 genome assembly). Analyses for enrichment at promoters (up to 3 kb before the transcription start site), exons and introns were performed using the CEAS program [64]. ChIP-chip data of young adult H3K4me3 and H3K36me3 marks [35], and the embryonic LEM-2 mark [18] are from modENCODE [65] (accession IDs modENCODE_3552, modENCODE_3559, modENCODE_2729, respectively). Embryonic LMN-1 DamID data [34] were obtained from the Gene Expression Omnibus (GEO; accession GSE37226). Gene sets used in tissue-specific analyses were defined previously and are listed in Table S2 in Additional file 2: muscle [66], intestine [67], ubiquitous, serpentine, germ line [68], and neuron [69].

### Definition of LMN-1- and EMR-1-associated domains

LMN-1- and EMR-1-associated domains were defined as previously described [18]. Briefly, we transformed positive and negative MA2C scores to +1 and -1, respectively. We averaged the binary values in 200-probe windows (approximately 10 kb), sliding one probe (50 bp offset) across the genome to identify windows with high average binary values. To have an estimation of the false positive windows, we performed the same procedure on a GFP::Dam/GFP::Dam MA2C normalized dataset. From this, we defined positive windows when a binary window value is ≥0.8, at which the false discovery rate is <5% (Figure S2 in Additional file 1). We then joined any overlapping (≥1 bp) windows to generate the LMN-1- and EMR-1-associated domains (Table S1 in Additional file 2).

The definition of chromosomal arms and chromosomal centers for comparison of embryonic and adult LMN-1 association profiles (Figure 2D) was based on the boundaries of LADs in adult nematodes. Coordinates of chromosome arms: chromosome I, 1 to 3,745,632 and 10,809,938 to 15,072,421; chromosome II, 1 to 4,708,341 and 11,877,168 to 15,279,323; chromosome III, 1 to 3,508,994 and 9,947,268 to 13,783,681; chromosome IV, 1 to 7,317,812 and 12176625 to 17,493,785; chromosome X, 1 to 4,191,936.

To define ‘EMR-1 only’ elements, we selected probes with a MA2C score ≥1 in EMR-1 DamID experiments and <0 in LMN-1 DamID experiments. We defined ‘EMR-1 only’ elements as the regions that contain at least 10 positive probes, each separated ≤500 bp from the neighboring positive probe (Table S1 in Additional file 2). We performed the same procedure to identify ‘LMN-1 only’ elements (Table S1 in Additional file 2).

### RNAi and RNA extraction

To simultaneously deplete EMR-1 and LEM-2, BN19 animals homozygous for the *lem-2(tm1582)* allele were subjected to *emr-1* RNAi-mediated knockdown. RNAi feeding bacteria were grown in LB at 37°C with agitation (180 rpm) until OD_600_ = 0.8 and then induced with 1 mM isopropyl β-D-1-thiogalactopyranoside (IPTG) during 19 h at 20°C. The RNAi bacteria expressed double-stranded RNA corresponding to either full-length *emr-1* cDNA or, as negative control, 185 bp of unrelated sequence from the L4440 empty plasmid. The next day, the bacteria were collected and resuspended in 1/20 initial volume of S-medium with 1 mM IPTG. Approximately 40,000 synchronized L1 nematodes obtained by hypochlorite treatment and hatching overnight at 20°C without food were grown in 50 ml of S-medium containing 5 ml of resuspended RNAi bacteria, 100 μg/ml ampicillin and 1 mM IPTG at 20°C during 53 h with continuous agitation (180 rpm). At this point the cultures accumulated non-gravid young adults. Nematodes were recovered, frozen immediately in liquid N_2_ and kept at -80°C until further processing. Part of the culture was grown for an additional day, which confirmed the expected embryonic lethal phenotype in the EMR-1 LEM-2 double depletion. RNA extraction was performed using 20 to 30 mg of nematodes. The nematode cuticle was ruptured by three rounds of freezing in liquid N_2_ and thawing at 30ºC and RNA was purified using the RNeasy Mini kit (QIAGEN).

### Expression profiling

Sequencing libraries were prepared using the TruSeq RNA sample preparation kit according to the manufacturer’s instructions (Illumina, San Diego, CA, USA). Briefly, polyA-containing mRNA was isolated from 1 μg of total RNA using poly-T oligo-attached magnetic beads and processed as described [70]. Pools of three indexed libraries were mixed (multiplexed) at equimolar ratios to yield a total oligonucleotide mix of 10 nM. Finally, the resulting libraries were sequenced on the Illumina Genome Analyzer IIx platform to generate 150 bp single-end reads. Three pooled indexed libraries were sequenced in each flow cell lane.

Raw RNA sequence data were processed [70] and aligned with the WS231 genome version using TopHat. Next, results were sorted and indexed using Samtools. Coverage vectors for each sample were extracted under R statistical environment using Rsamtools library. The *C. elegans* GTF annotation from Ensembl was applied as reference for the exon genomic ranges, parsed into R data structures. Exon expression was calculated as the median coverage for every exon genomic range as stated by annotation. Exon expression data from our nine different samples (three N2, two BN19, two BN20, and two BN24) were normalized by quantile normalization and locus expression was defined as the median of all the expression hits from distinct exons annotated to a single locus. Library siggenes in R was applied to select the differentially expressed loci using the false discovery rate-based SAM method [71]. Genes presenting a Δ value >4 were considered to be significantly altered (Table S3 in Additional file 2). Resulting data are available with accession numbers summarized in Table S4 in Additional file 2. For the analysis of *emr-1(gk119)* and *lem-2 (tm1582)* mutants, the sets of genes expressed in different tissues were the same as in the DamID analysis (Table S2 in Additional file 2).

### Gene Ontology analysis

GO analyses were performed using DAVID [72] and only the ‘Biological Processes’ tree was used in our study. For the study of DamID ‘EMR-1 only’ elements, as they were enriched in promoters and regulatory regions, the closest but not overlapping genes were selected. In the other cases, we selected genes with ≥50% of the open reading frame included within the domains.

### Aldicarb sensitivity experiments

Sensitivity to aldicarb was determined by assaying the time course of the onset of paralysis following acute exposure of a population of animals to this drug. In each experiment 20 to 25 young adults were placed on freshly made drug plates (1 mM aldicarb; Fluka Sigma-Aldrich ref. 33386) and prodded every 10 minutes over a 2 h period to determine if they retained the ability to move. Nematodes that failed to respond to touch were classified as paralyzed. Each experiment was repeated four times by two independent experimenters.

### Data access

DamID and RNA sequencing data performed in this study are available from GEO [73] accession IDs GSE44188 and GSE44682, respectively (described in Table S4 in Additional file 2).

## Abbreviations

bp: base pair; ChIP: chromatin immunoprecipitation; DamID: DNA adenine methyltransferase identification; EAD: EMR-1-associated domain; EDMD: Emery-Dreifuss muscular dystrophy; ESC: embryonic stem cell; gDNA: genomic DNA; GEO: Gene Expression Omnibus; GO: Gene Ontology; INM: inner nuclear membrane; IPTG: isopropyl β-D-1-thiogalactopyranoside; LAD: LMN-1-associated domain; NE: nuclear envelope; ONM: outer nuclear membrane; PCR: polymerase chain reaction; RNAi: RNA interference.

## Competing interests

The authors declare that they have no competing interests.

## Authors’ contributions

CGA performed all experiments and analyzed the data. KI assisted in data analysis and CA assisted in aldicarb assays. AdL, MI and JC analyzed RNA samples. KI and JDL helped to draft the manuscript. CGA and PA conceived the study, designed experiments and wrote the paper. All authors read and approved the final manuscript.

## Supplementary Material

Additional file 1: Figure S1 DamID fusion proteins localize properly at the NE. **Figure S2:** quality assessment of LMN-1 and EMR-1 DamID data. **Figure S3:** LMN-1 and EMR-1 associate with the ends of chromosomes and silenced DNA. **Figure S4:** analysis of ‘EMR-1 only’ and ‘LMN-1 only’ elements. **Figure S5:** differential expression of EMR-1 and LEM-2. **Figure S6:** genes expressed in different tissues show distinct association with EMR-1 and LMN-1. **Figure S7:** analysis of LMN-1 DNA association in wild type and *emr-1* and *lem-2* mutants. **Figure S8:** expression analyses of genes associated with LMN-1 and EMR-1. Click here for file

Additional file 2: Table S1 The coordinates for EADs, LADs, ‘EMR-1 only’ elements and ‘LMN-1 only’ elements. **Table S2:** genes that are expressed in specific tissues. **Table S3:** RNA sequencing data. **Table S4:** GEO accession numbers. Click here for file

Additional file 3 **Tables S5 to S10:** Information on significant GO categories. Click here for file

Additional file 4 Supplementary Materials and methods. Click here for file
