# Supplementary material for: Genome-wide analysis links emerin to neuromuscular junction activity in Caenorhabditis elegans
Source: Genome Biol. 2014 Feb 3;15(2):R21. doi: 10.1186/gb-2014-15-2-r21 (PMC4053756; doi:10.1186/gb-2014-15-2-r21)
Supplement: Additional file 1: Figure S1 — DamID fusion proteins localize properly at the NE. Figure S2: quality assessment of LMN-1 and EMR-1 DamID data. Figure S3: LMN-1 and EMR-1 associate with the ends of chromosomes and silenced DNA. Figure S4: analysis of ‘EMR-1 only’ and ‘LMN-1 only’ elements. Figure S5: differential expression of EMR-1 and LEM-2. Figure S6: genes expressed in different tissues show distinct association with EMR-1 and LMN-1. Figure S7: analysis of LMN-1 DNA association in wild type and emr-1 and lem-2 mutants. Figure S8: expression analyses of genes associated with LMN-1 and EMR-1. [file gb-2014-15-2-r21-S1.pdf]

Supplemental Figures. González-Aguilera et al.

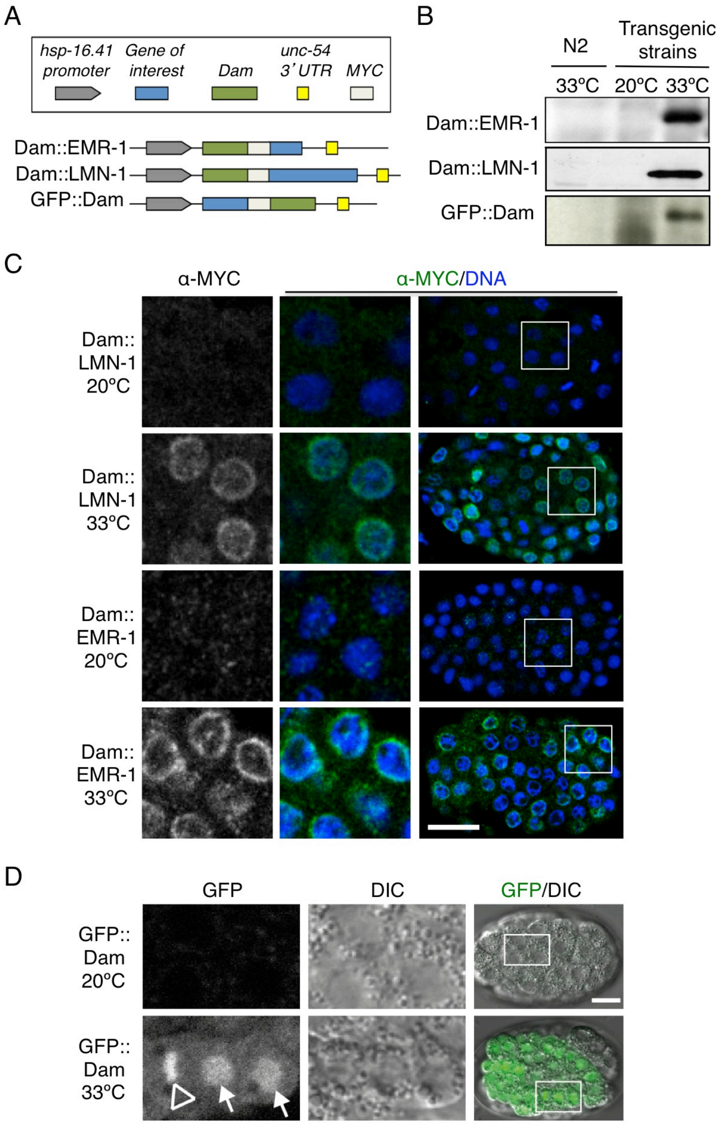

Figure S1. DamID fusion proteins localize properly at the NE. (*A*) Schematic representation of Dam-fusion constructs. Chimeric proteins were expressed under the control of the *hsp-16.41* heat-shock promoter and the *unc-54* 3'UTR. A MYC epitope was inserted between the proteins of interest and Dam to facilitate detection of the chimera. Not drawn to scale. (*B-D*) Embryos were obtained from nematodes incubated constantly at 20ºC or heat-shocked 1h at 33ºC and left to recover for 2h at 20ºC. (*B*) Western-blot analysis of embryos from wild type (N2) and transgenic strains expressing Dam::EMR-1, Dam::LMN-1 or GFP::Dam fusion proteins probed with anti-MYC antibody. (*C*) Dam::LMN-1 and Dam::EMR-1 expressing embryos were fixed and stained with anti-MYC antibody (green) and Hoechst 33258 to visualize chromatin (blue). Localization to the NE is observed for both fusion proteins after heat shock. (*D*) GFP::Dam expressing embryos were analyzed by live confocal microscopy. Arrows point to nuclei, whereas an open triangle indicates GFP::Dam associated with metaphase chromosomes. Boxed regions in merge images (right) are shown at higher magnification to the left. Scale bars, 10μm.

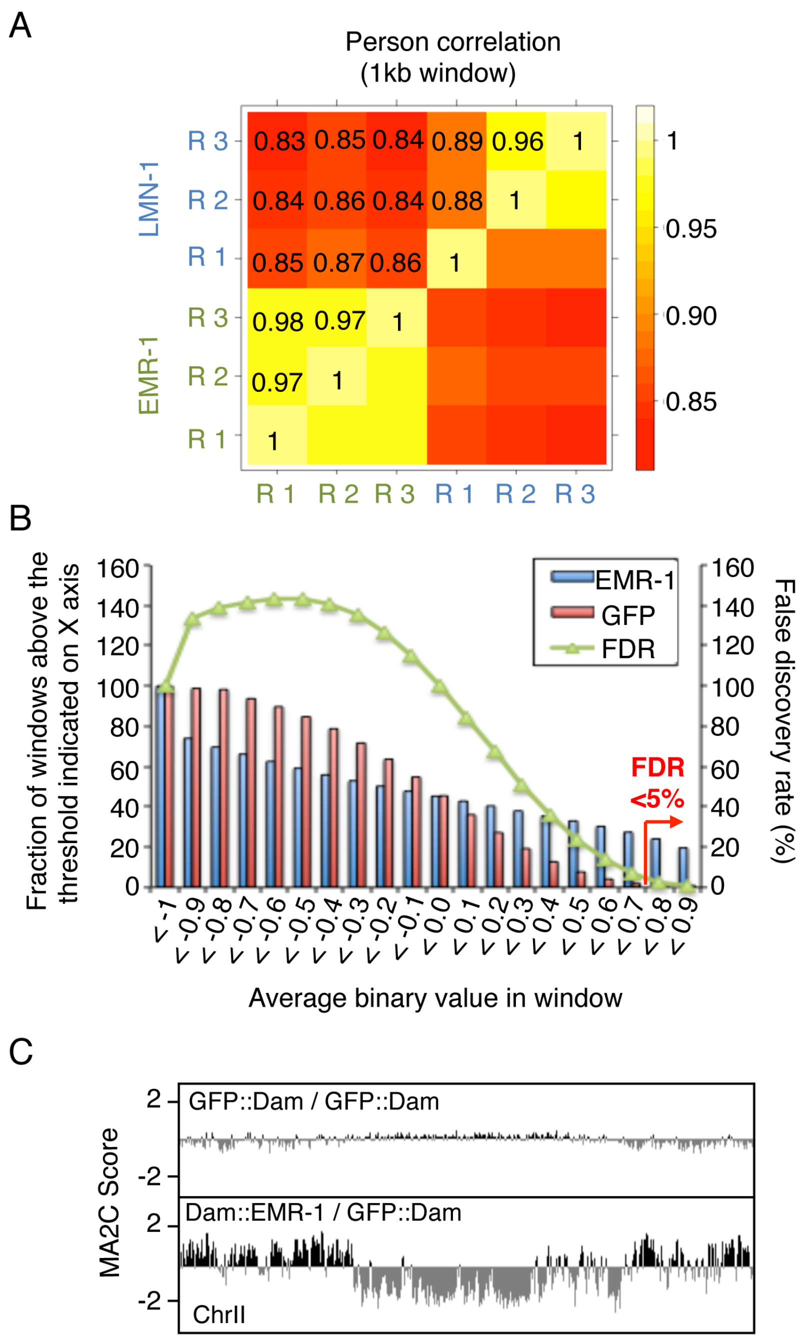

Figure S2. Quality assessment of LMN-1 and EMR-1 DamID data. (*A*) Heatmap of Pearson correlation coefficients for the three replicates of Dam::LMN-1 and Dam::EMR-1 DamID experiments. Correlations were calculated based on MA2C scores of all probes on the microarrays (1 kb window averaged). (*B*) Estimation of the False Discovery Rate (FDR) for EMR-1 DamID domain calling. The *x* axis bins windows in DamID (200 probes) according to threshold for average binary values within windows (Material and methods). The right *y* axis indicates FDR of each bin, which was estimated by the proportion of the number of control GFP::Dam windows to the number of Dam::EMR-1 windows. (*C*) Genome browser views of MA2C scores obtained for chromosome II when normalizing Dam::EMR-1 versus GFP::Dam (bottom) or GFP::Dam versus GFP::Dam (top)(1 replicate each). Lack of enriched regions in the GFP::Dam/GFP::Dam control indicates that the identified EMR-1-associated domains are specific for the Dam::EMR-1 protein.

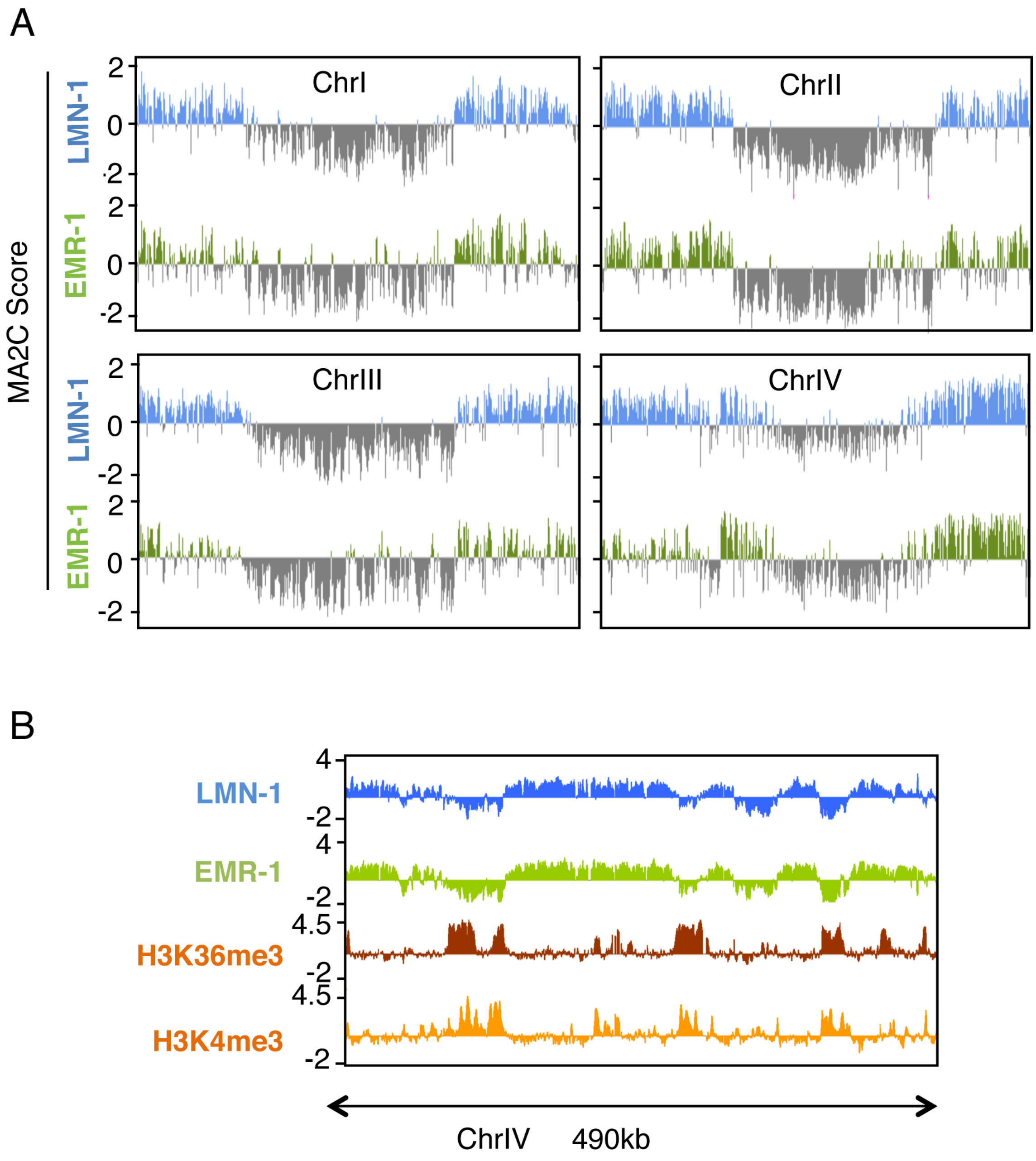

Figure S3. LMN-1 and EMR-1 associate with the ends of chromosomes and silenced DNA. (*A*) Regions enriched for Dam::LMN-1 and Dam::EMR-1 in chromosomes I, II, III and IV in adult worms. For each track, the average MA2C score probe signal of three independent biological replicates is plotted. (*B*) Representative pattern in chromosome IV illustrating that DamID LMN-1 and EMR-1 domains are devoid of H3K36me3 and H3K4me3, two histone marks linked to transcriptional activation. ChIP-chip data for histone marks were obtained from [35].

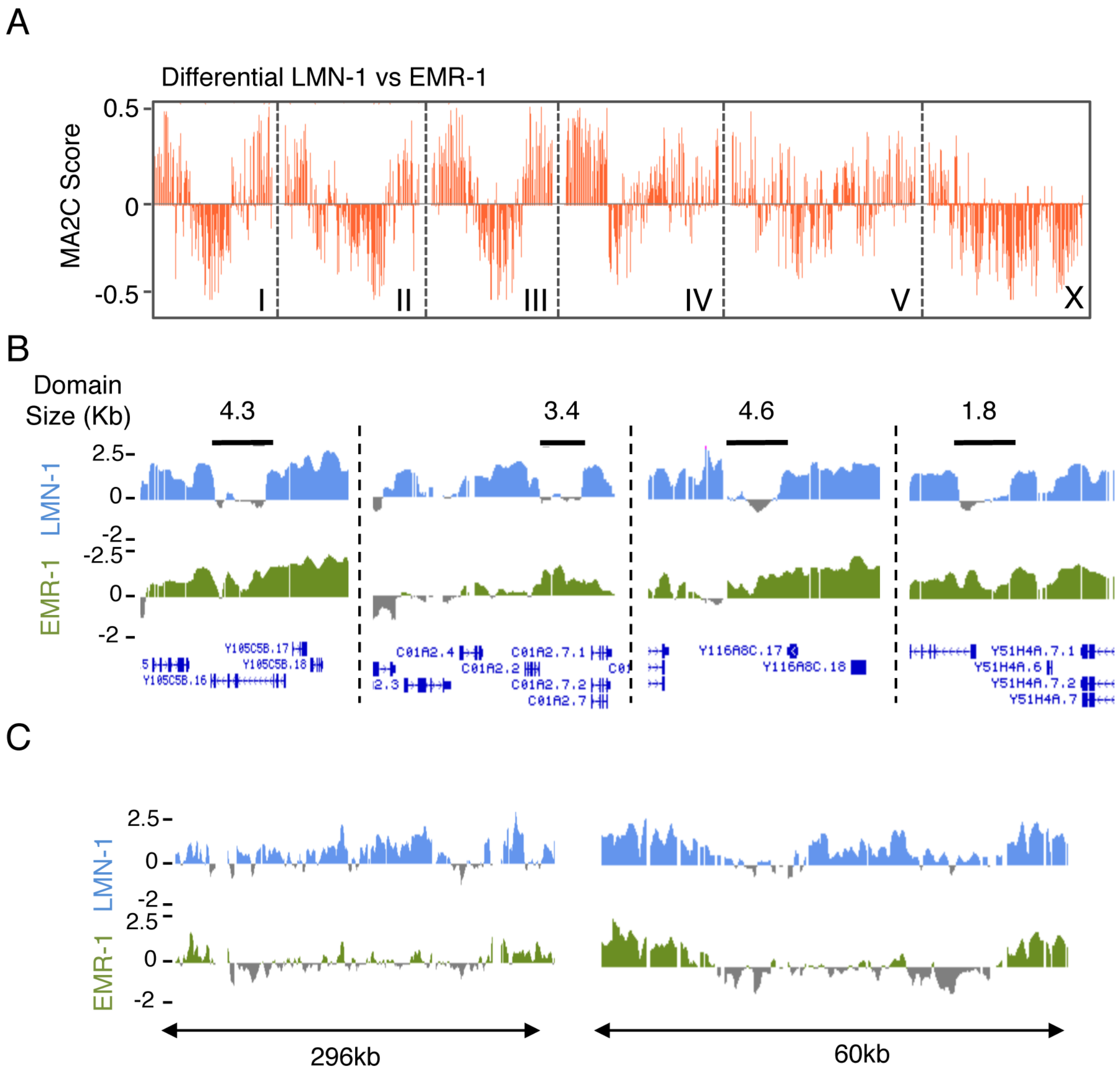

Figure S4. Analysis of 'EMR-1 only' and 'LMN-1 only' elements. (*A*) Differential profile of quantile normalized Dam::LMN-1 versus Dam::EMR-1 data in adult worms. Positive values reflect MA2C scores LMN-1>EMR-1; negative scores LMN-1<EMR-1. (*B*) Examples of 'EMR-1 only' elements indicated with horizontal black lines; sizes are in kb. Genes are indicated below in dark blue. (*C*) Examples of 'LMN-1 only' elements. These elements are included in large areas with reduced EMR-1 occupancy.

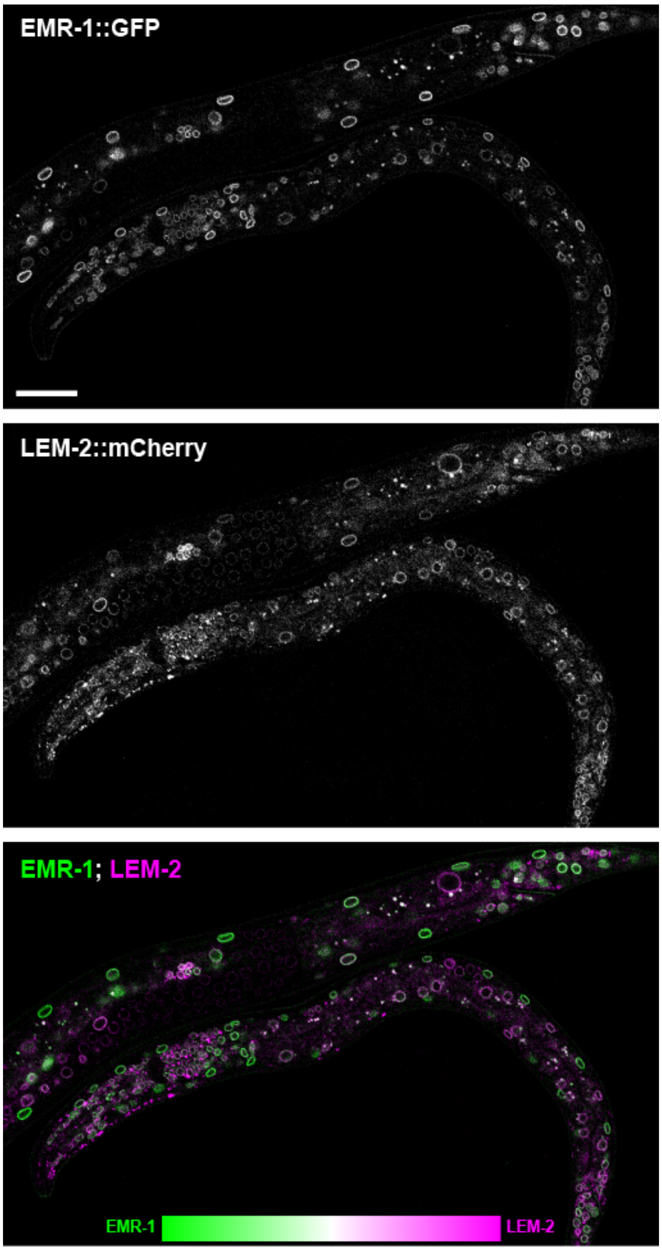

Figure S5. Differential expression of EMR-1 and LEM-2. Live imaging of animals expressing EMR-1::GFP and LEM-2::mCherry from single-copy transgenes and under control of the *emr-1* and *lem-2* promoter, respectively. Both genes are expressed in the NE in most if not all cells, but with considerable variation between tissues as reflected by different intensities in the two upper panels. In the merged image green and magenta represent high and low EMR-1::GFP to LEM-2::mCherry signal ratio, respectively. Note that these ratios reflect arbitrary fluorescence values and do not necessarily correspond to EMR-1 being more or less abundant than LEM-2.

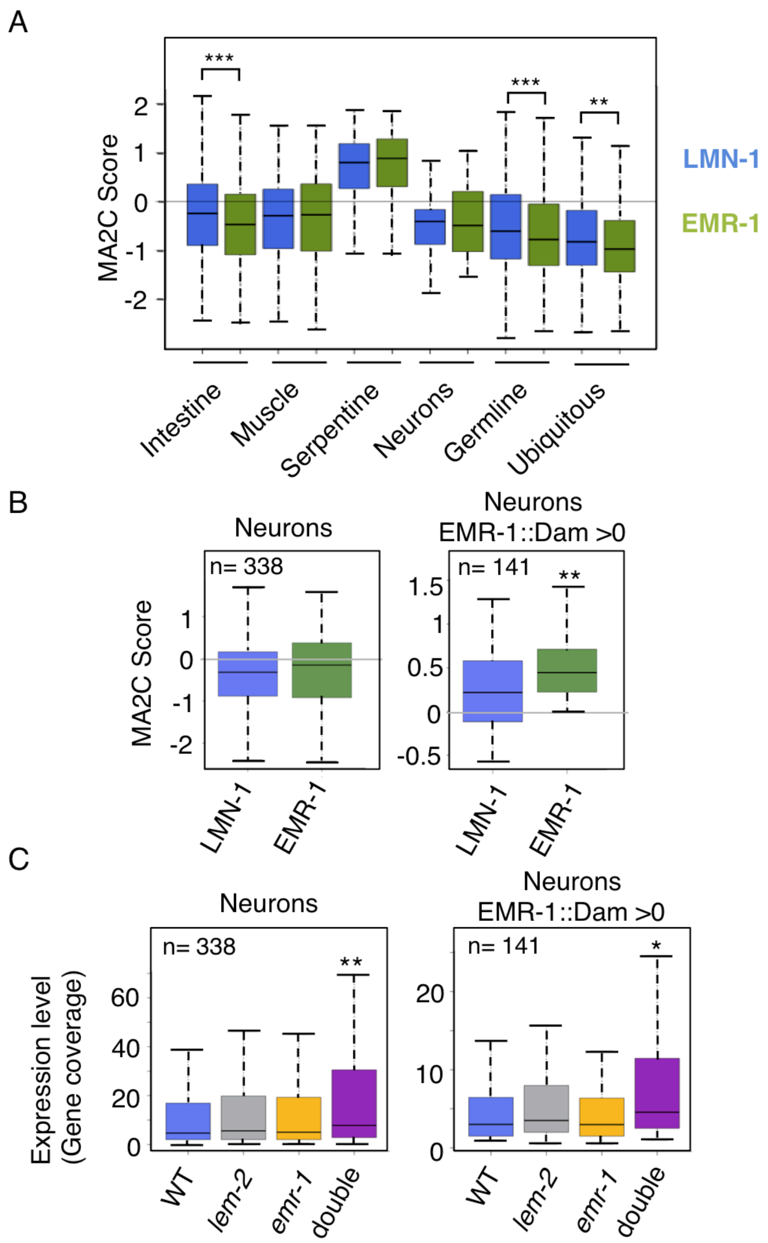

Figure S6. Genes expressed in different tissues during adult and larval stages show distinct association to EMR-1 and LMN-1. *(A)* LMN-1 (blue) and EMR-1 (green) occupancy for various gene sets defined on expression data in adult (Table S2 in Additional file 1). Number of genes in each class: intestine, 1818, muscle, 886, serpentine, 386, neuronal 46, germline, 2650, ubiquitous, 1168. Boxplots show the range of MA2C score averaged over gene bodies. *(B)* LMN-1 (blue) and EMR-1 (green) occupancy for 338 pan-neuronal genes defined from expression data in larval stages [41]. These genes were not expressed in any other tissue. Left panel shows occupancy for all 338 genes; right panel shows occupancy for 141 genes associated to EMR-1::Dam during adulthood. *(C)* Expression of the same genes described in *B* in wild type, *lem-2(tm1582), emr-1(gk119)* and *emr-1(RNAi) lem-2(tm1582)* (double) animals. Expression is measured as the median coverage of exons per gene. Boxes indicate the 25th and 75th percentiles and lines in the boxes indicate medians. Whiskers indicate the lowest and the highest data points within 1.5x interquartile range from the box. Wilcoxon rank sum test was used for the statistical analysis (*$p<0.05$, **$p<10^{-4}$, ***$p<10^{-15}$).

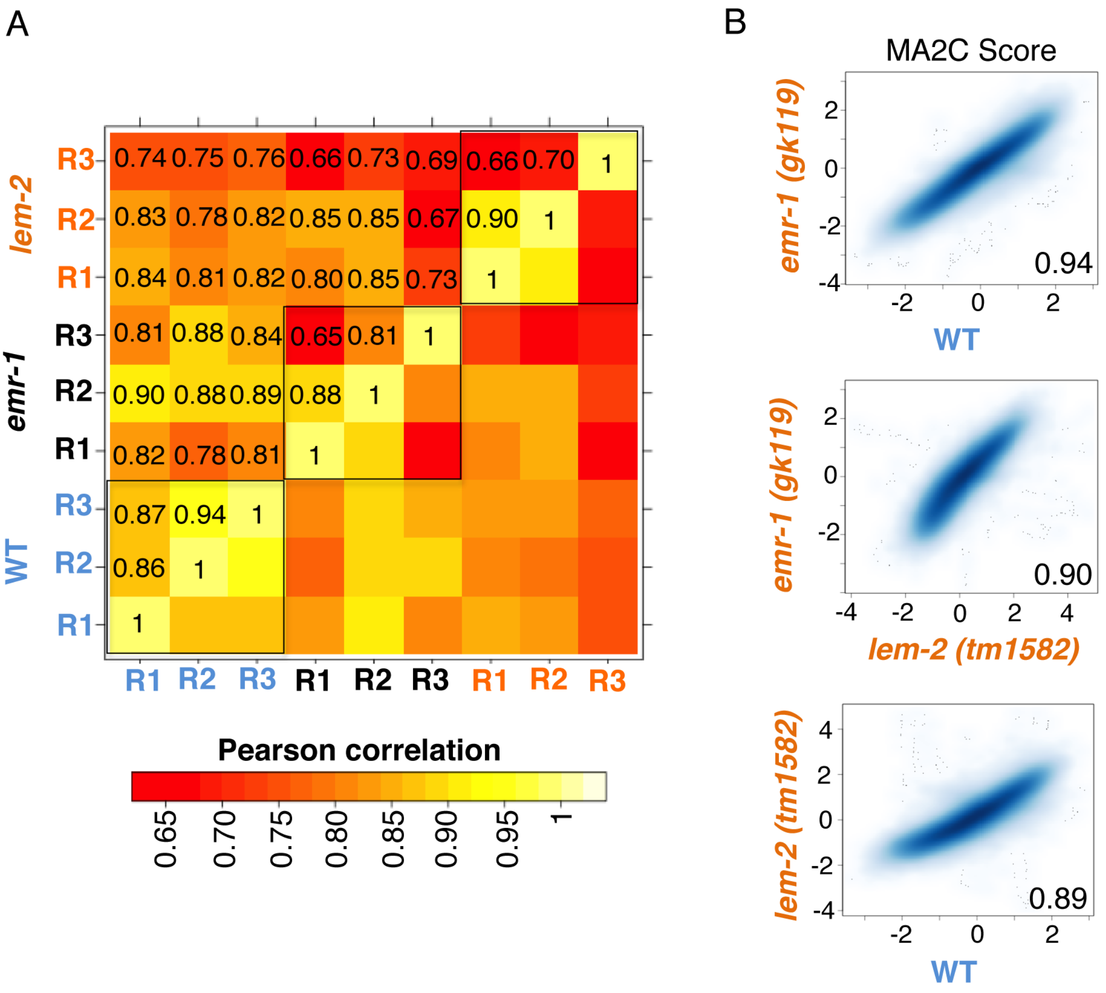

Figure S7. Analysis of LMN-1 DNA association in wild type, *emr-1* and *lem-2* mutants. (*A*) Heatmap of Pearson correlation coefficients for the three replicates of Dam::LMN-1 DamID experiments performed in wild type, *emr-1(gk119)* and *lem-2(tm1582)* mutants. Correlations were calculated based on MA2C scores of all probes on the microarrays. (*B*) Genome-wide correlation plot (probe level) of LMN-1 DNA association in wild type and *emr-1(gk119)* (top), wild type and *lem-2(tm1582)* (bottom) and *emr-1(gk119)* and *lem-2(tm1582)* (middle). The signal represents the average of three independent replicates for each background. Numbers in the lower right corners indicate Pearson correlation coefficients.

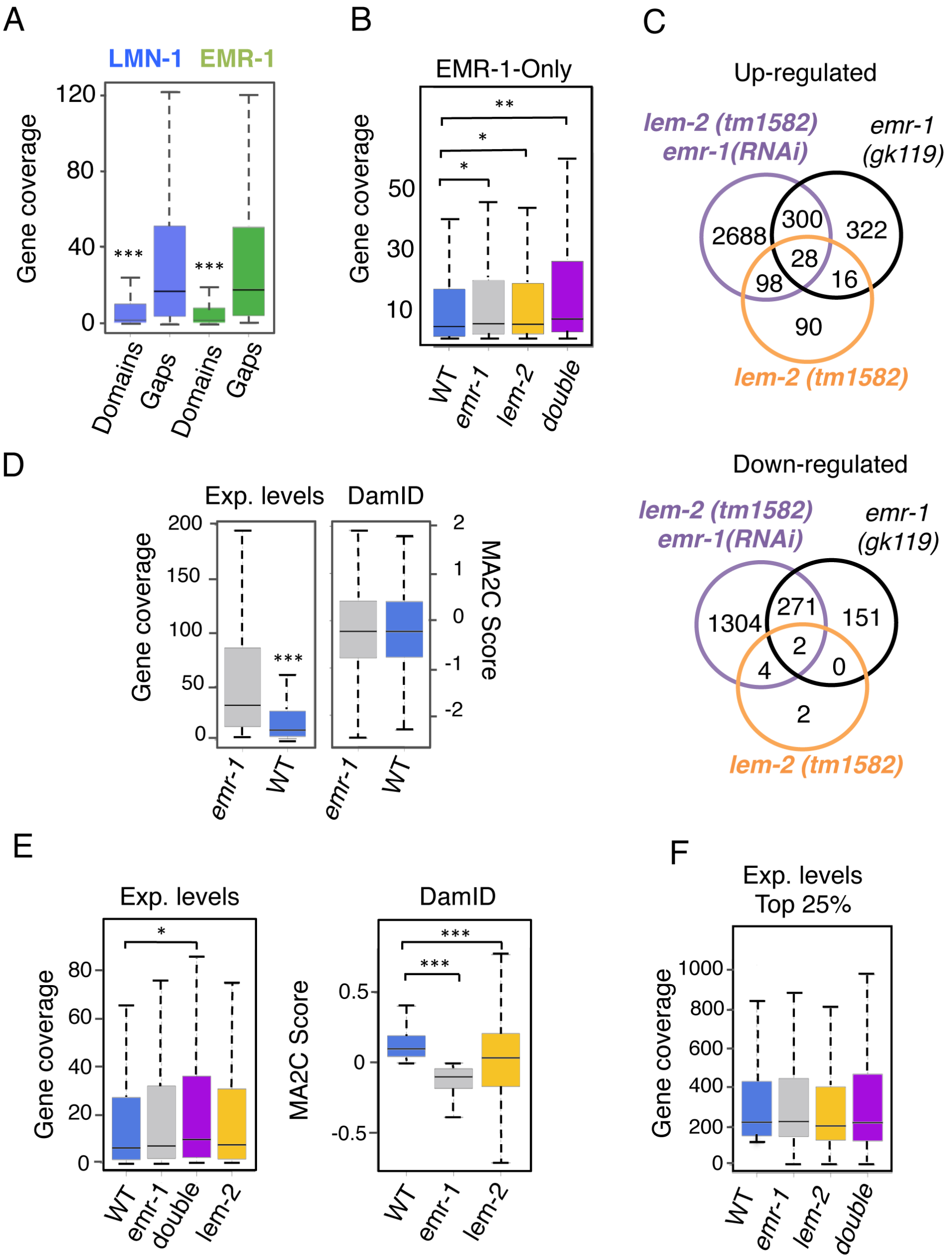

Figure S8. Expression analyses of genes associated with LMN-1 and EMR-1. (*A*) Expression of transcripts included in LADs, EADs and their respective gaps, measured as the median coverage of exons per gene. (*B*) Comparison of the expression of transcripts associated to 'EMR-1 only' elements in wild type (WT), *lem-2(tm1582), emr-1(gk119)* and *emr-1(RNAi) lem-2(tm1582)* (double) animals. Significant increase in gene expression in single and double mutants was observed. (*C*) Venn diagrams showing the number of transcripts up- or down-regulated in the backgrounds described before. (*D*) Genes that are up-regulated in *emr-1(gk119)* mutants (left graph) have similar association with LMN-1 in wild type and *emr-1* mutants (right graph). (*E*) Transcripts included in LADs that lose their association with the NL in *emr-1(gk119)* mutants (right graph) are significantly up-regulated in *emr-1(RNAi) lem-2(tm1582)* (double) animals (left graph). (*F*) Comparison of the top 25% most expressed genes in wild type, *lem-2(tm1582), emr-1(gk119) and lem-2(tm1582) emr-1(RNAi)* backgrounds. There were no significant differences in the expression of these genes between wild type and mutant strains. Boxplots as described in Figure S6. Wilcoxon rank sum test was used for the statistical analysis (* $p<10^{-2}$, ** $p<10^{-8}$, *** $p<10^{-13}$).
