# Supplementary material for: Genome-wide analysis links emerin to neuromuscular junction activity in Caenorhabditis elegans
Source: Genome Biol. 2014 Feb 3;15(2):R21. doi: 10.1186/gb-2014-15-2-r21 (PMC4053756; doi:10.1186/gb-2014-15-2-r21)
Supplement: Additional file 4 — Supplementary Materials and methods. [file gb-2014-15-2-r21-S4.pdf]

Supplemental Material. González-Aguilera C. et al.

Plasmids

Plasmid **pBN65** ($P_{hsp\text{-}16.41}$*::hsp16.41p::dam::myc::lmn-1*) was made inserting a *lmn-1 NgoM*IV/*Xma*I fragment amplified from genomic DNA with primers 5'CGC GCG CCG GCT CAT CTC GTA AAG GTA CT 3' + 5' CGC GCC CCG GGT TAC ATG ATG GAA CAA CGA TC 3' into *NgoM*IV of pBN61. Plasmid **pBN61** ($P_{hsp\text{-}16.41}$*::dam::myc*_w/o STOP) contains a *Xho*I/*NgoM*IV fragment amplified from pNDamMYC with primers 5'CGC GCC TCG AGA AAA ATG AAG AAA AAT CGC GCT 3' + 5' CGC GCG CCG GCC AGA TCC TCT TCA GAG AT3' into *Xho*I/*NgoM*IV of pBN16. Plasmid **pBN16** ($P_{hsp\text{-}16.41}$*::gfp::unc-54 3'UTR*) was made amplifying the $P_{hsp\text{-}16.41}$*::gfp::unc-54 3'UTR* in two parts with primers 5' ATG CGG CCG CCA AGC TTG CAT GCC TGC 3' + 5' GCA ACG CGT GGA GCT AGC GCA TCG CGA AGC TCC GCA TCG GCC GCT GT 3', and 5'GCA GCG GCG AAA GAA GC 3' + 5'GCA ACG CGT GGA GCT AGC GCA TCG CGA AGC TCC GCA TCG GCC GCT GT 3' from pBN4, cut with *Mlu*I, ligated, cut with *Not*I/*Spe*I and inserted into pBN8 *Not*I/*Spe*I site. Plasmid **pBN4** ($P_{hsp\text{-}16.41}$*::gfp*) was made inserting a *BamH*I/*Sac*I GFP fragment from pJH4.52 into pPD49.83. Plasmid **pBN8** was a modification of pCFJ151 by insertion of longer polylinker formed by primer duplex of 5'TCG AGG AGC CGG CAT GCT AGC ATG CGG CCG CTA CCT GCA 3' and 5'GGT AGC GGC CGC ATG CTA GCA TGC CGG CTC C 3'. Plasmid **pBN67** ($P_{hsp\text{-}16.41}$*::gfp::myc::dam*) contains a *NgoM*IV/*Bgl*II fragment of pBN63 into *NgoM*IV/*Bgl*II fragment of pBN16. Plasmid **pBN63** ($P_{hsp\text{-}16.41}$*::emr-1::myc::dam*) was made inserting a *NgoM*IV/*Bgl*II fragment amplified from pCMYCDam with primers 5'CGC GCG CCG GCG AAC AGA AAC TCA TCT CT 3' + 5'CGC GCA GAT CTT TAT TTT TTC GCG GGT GAA AC 3' into *NgoM*IV/*Bgl*II of pBN62. Plasmid **pBN62** ($P_{hsp\text{-}16.41}$*::emr-1*) contains a *Xho*I/*NgoM*IV fragment amplified from pPAG7 with 5'CGC GCC TCG AGA AAA ATG GAC GTC TCC CAG CTG 3' and 5'CGC GCG CCG GCA ATA GTA TCC TCC GGA TT 3' into *Xho*I/*NgoM*IV of pBN16. Plasmid **pBN79** ($P_{hsp\text{-}16.41}$*::Dam::MYC::emr-1)* contains a *Bgl*II/*Nhe*I fragment of plasmid pSpark-emr inserted into BglII/NheI site of plasmid pBN61. Plasmid **pSpark-emr** contains *emr-1* gDNA fragment amplified by PCR with primers 5' CGC GCA GAT CTG ACG TCT CCC AGC TGA CAG A 3' and 5' CGC GCG CTA GCT TAA ATA GTA TCC TCC GGA T 3' inserted in plasmid pSpark I (Canvax Biotech SL.). Plasmid **pBN37** contains the *lem-2* gene including 1.1kb of sequence upstream of the translational start site, 0.6kb of sequence downstream of the stop codon and mCherry inserted into an engineered BsrGI site immediately before the stop codon. Similarly, plasmid **pBN103** contain the *emr-1* gene with 1.6kb upstream and 0.3kb downstream sequences as well as GFP inserted into an engineered BsrGI site immediately before the stop codon. Further details are available upon request. Plasmids pJL43, pCFJ90, pCFJ104, pCFJ151 (Frokjaer-Jensen et al. 2008), pNdamMYC, pCMYCDam (van Steensel and Henikoff 2000), pPAG7(Askjaer et al. 2002) pJH4.52 (Strome et al. 2001), pPD49.83 (Mello and Fire 1995) and pBN1 (Ródenas et al., 2012) were described previously.

Strains

BN19 (*lem-2(tm1582)* II) and **BN20** (*emr-1(gk119)* I) were generated by outcrossing original deletion strains *tm1582* from the National Bioresource Project and VC237 from the International *C. elegans* Gene Knockout Consortium, respectively, with the *C. elegans* Bristol wild type strain N2 seven times. Strain **BN24** (*emr-1(gk119)*

I/hT2(I;III); *lem-2(tm1582)* II) was obtained by crossing BN19 with outcrossed hT2/+ (VC699 crossed twice with N2) followed by mating with BN20.

Strains **BN195** (*bqSi195*[pBN65(*unc-119(+)* $P_{hsp-16.41}$*::dam::myc::lmn-1*)] II), **BN196** (*bqSi196*[pBN67(*unc-119(+)* $P_{hsp-16.41}$*::gfp::myc::dam*)] II), **BN218** (*bqSi218*[pBN79(*unc-119(+)* $P_{hsp-16.41}$*::dam::myc::emr-1*)] II), and **BN235** (*bqSi235*[pBN103(*unc-119(+)* P_{emr-1}*::emr-1::GFP*)] II) were constructed injecting the plasmids pBN65, pBN67, pBN79, and pBN103, respectively, together with transposase and transformation markers (pJL43.1, pCFJ90, pCFJ104 and pBN1) into strain **EG4322** (*ttTi5605* II; *unc-119(ed3)* III) (Frokjaer-Jensen et al. 2008). Strain **BN242** (*bqSi242*[pBN37(*unc-119(+)* P_{lem-2}*::lem-2::mCherry*)] IV) was constructed injecting the plasmid pBN37 together with transposase and transformation markers (pJL43.1, pBN40, pBN41 and pBN2) into strain **EG5003** (*unc-119(ed3)* III; *cxTi10882* IV.) (Frokjaer-Jensen et al. 2008). Strains carrying the integrations were outcrossed twice with N2. Single insertions of DamID constructs at the correct location were verified by Southern blot and PCR analyses.

Strains **BN198** (*lem-2(tm1582) bqSi196*[pBN67*(unc-119(+)* $P_{hsp-16.41}$*::gfp::myc::dam)*] II) and **BN202** (*emr-1(gk119)* I*; bqSi196*[pBN67*(unc-119(+)* $P_{hsp-16.41}$*::gfp::myc::dam)*] II) were constructed crossing BN196 with BN19 and BN20, respectively. Strains **BN199** (*lem-2(tm1582) bqSi195*[pBN65*(unc-119(+)* $P_{hsp-16.41}$*::dam::myc::lmn-1*)] II) and **BN203** *(emr-1(gk119)* I*; bqSi195*[pBN65*(unc-119(+)* $P_{hsp-16.41}$*::dam::myc::lmn-1*)] II) were constructed crossing BN195 with BN19 and BN20, respectively. Strain **BN243** (P_{emr-1}*::emr-1::GFP* II; P_{lem-2}*::lem-2::mCherry* IV) was constructed crossing pBN235 with pBN242.

References

Askjaer P, Galy V, Hannak E, Mattaj IW. 2002. Ran GTPase cycle and importins alpha and beta are essential for spindle formation and nuclear envelope assembly in living Caenorhabditis elegans embryos. *Molecular biology of the cell* **13**(12): 4355-4370.

Frokjaer-Jensen C, Davis MW, Hopkins CE, Newman BJ, Thummel JM, Olesen SP, Grunnet M, Jorgensen EM. 2008. Single-copy insertion of transgenes in Caenorhabditis elegans. *Nature genetics* **40**(11): 1375-1383.

Mello C, Fire A. 1995. DNA transformation. *Methods in cell biology* **48**: 451-482.

Strome S, Powers J, Dunn M, Reese K, Malone CJ, White J, Seydoux G, Saxton W. 2001. Spindle dynamics and the role of gamma-tubulin in early Caenorhabditis elegans embryos. *Mol Biol Cell* **12**(6): 1751-1764.

van Steensel B, Henikoff S. 2000. Identification of in vivo DNA targets of chromatin proteins using tethered dam methyltransferase. *Nature biotechnology* **18**(4): 424-428.
